# Transcriptomic Profile of Whole Blood Cells from Elderly Subjects Fed Probiotic Bacteria *Lactobacillus rhamnosus* GG ATCC 53103 (LGG) in a Phase I Open Label Study

**DOI:** 10.1371/journal.pone.0147426

**Published:** 2016-02-09

**Authors:** Gloria Solano-Aguilar, Aleksey Molokin, Christine Botelho, Anne-Maria Fiorino, Bryan Vinyard, Robert Li, Celine Chen, Joseph Urban, Harry Dawson, Irina Andreyeva, Miriam Haverkamp, Patricia L. Hibberd

**Affiliations:** 1 Diet, Genomics, and Immunology Laboratory, Beltsville Human Nutrition Research Center, Agricultural Research Service, United States Department of Agriculture, Beltsville, Maryland, United States of America; 2 Division of Global Health, Massachusetts General Hospital, Boston, Massachusetts, United States of America; 3 Statistics Group, Northeast Area, Agricultural Research Service, United States Department of Agriculture, Beltsville, Maryland, United States of America; 4 Animal Genomics and Improvement Laboratory, Agricultural Research Service, United States Department of Agriculture, Beltsville, Maryland, United States of America; National University of Singapore, SINGAPORE

## Abstract

We examined gene expression of whole blood cells (WBC) from 11 healthy elderly volunteers participating on a Phase I open label study before and after oral treatment with *Lactobacillus rhamnosus* GG-ATCC 53103 (LGG)) using RNA-sequencing (RNA-Seq). Elderly patients (65–80 yrs) completed a clinical assessment for health status and had blood drawn for cellular RNA extraction at study admission (Baseline), after 28 days of daily LGG treatment (Day 28) and at the end of the study (Day 56) after LGG treatment had been suspended for 28 days. Treatment compliance was verified by measuring LGG-DNA copy levels detected in host fecal samples. Normalized gene expression levels in WBC RNA were analyzed using a paired design built within three analysis platforms (edgeR, DESeq2 and TSPM) commonly used for gene count data analysis. From the 25,990 transcripts detected, 95 differentially expressed genes (DEGs) were detected in common by all analysis platforms with a nominal significant difference in gene expression at Day 28 following LGG treatment (FDR<0.1; 77 decreased and 18 increased). With a more stringent significance threshold (FDR<0.05), only two genes (*FCER2* and *LY86*), were down-regulated more than 1.5 fold and met the criteria for differential expression across two analysis platforms. The remaining 93 genes were only detected at this threshold level with DESeq2 platform. Data analysis for biological interpretation of DEGs with an absolute fold change of 1.5 revealed down-regulation of overlapping genes involved with *Cellular movement*, *Cell to cell signaling interactions*, *Immune cell trafficking and Inflammatory response*. These data provide evidence for LGG-induced transcriptional modulation in healthy elderly volunteers because pre-treatment transcription levels were restored at 28 days after LGG treatment was stopped. To gain insight into the signaling pathways affected in response to LGG treatment, DEG were mapped using biological pathways and genomic data mining packages to indicate significant biological relevance.

***Trial Registration*:** ClinicalTrials.gov NCT01274598

## Introduction

*Lactobacillus rhamnosus* GG (LGG) isolated from human intestine is a well characterized strain shown to have antimicrobial effects against enteric bacterial pathogens and rotavirus [1] respiratory viruses such as respiratory syncytial virus (RSV) [2], rhinovirus infections [3] and influenza [4,5,6]. Immune modulating mechanisms attributed to probiotic bacteria like LGG have been based principally on *in vitro* cell culture models [4,7], some recently summarized *in vivo* models [1,8] and limited controlled intervention studies in humans [9]. However, there has been no convincing clinical demonstration of LGG-induced immune modulation in human patients given prolonged probiotic consumption [1].

Current evidence indicates that *Lactobacillus rhamnosus* (*L*. *rhamnosus*) can ameliorate intestinal injury and inflammation caused by various stimuli. *L*. *rhamnosus* species can specifically exert protective activity against lipopolysaccharide (LPS) induced inflammatory damage in animal models [10,11] or cells lines by blocking TNFα- and LPS-induced IL-8 activation [12,13]. It has also been reported that probiotic derived factors can reverse pathogen-induced inflammation. LGG modulates LPS-induced inflammation by decreasing the activation of pro-inflammatory transcription factor NF-Kb and IL-6 secretion, while inducing the anti-inflammatory cytokine IL-10 [10].

As one of the most experimentally and commercially used probiotics, LGG, was originally isolated from human intestine and has been extensively characterized [14]. *L*. *rhamnosus* is among the largest of the lactic acid bacteria that has the ability to persist in human intestinal mucosa displaying functional pili and producing bacteriocins [9]. The health benefits of LGG have been demonstrated in human feeding studies with normal populations or subjects suffering from gastrointestinal disorders and allergies [9,15].

Research using *in vitro* and *in vivo* animal models have been used to characterize the mechanisms employed by LGG to modulate epithelial barrier function [16], stimulate specific immune cell function[8], and utilize bacteria-host crosstalk to displace pathogenic bacteria from intestinal compartments [17]. However, no study has comprehensively evaluated the effect of continuous LGG consumption on changes in human whole blood cell transcriptome as an indicator of safety and immune modulating activity. The primary aim of this Phase I open label study was to provide information on adverse events that may occur in healthy elderly volunteers receiving LGG administered twice a day for 28 days [18]. The secondary aim as described in this manuscript was to evaluate potential mechanisms of action of LGG in the healthy elderly by studying their immunologic responses to consumption of LGG for 28 days.

## Methods

### Ethics Statement

This study was approved by the Partners Institutional Review Board (IRB 2010P001695) and was registered at ClinicalTrials.gov (NCT01274598). An Independent Data Safety Monitoring Board reviewed the protocol prior to initiation and throughout study. In addition, the study was monitored by the Center for Biologics Evaluation and Research (CBER) from FDA under IND 14377 and the National Institutes of Health (NIH) Office of Clinical and Regulatory Affairs (OCRA) and National Center for Complementary and Integrative Health (NCCIH). The protocol for this trial and supporting CONSORT checklist are available as supporting information S1 Fig and S1 Table. All data is available for public access through the database of Genotypes and Phenotypes (dbGaP) (www.ncbi.nlm.nih.gov/gap) accession phs000928.v1.p1.

### Study design

**Fig 1:**
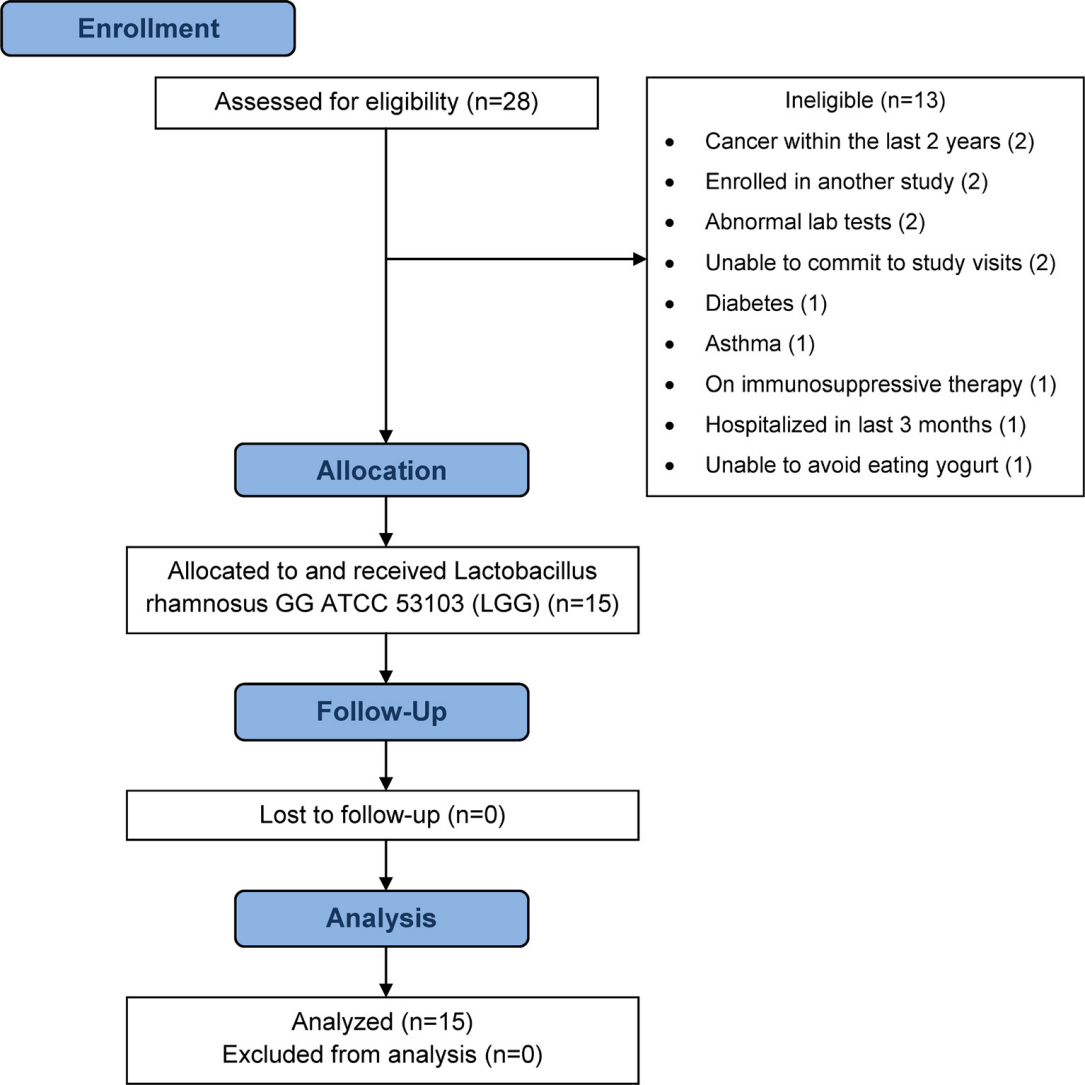
Participant flow diagram.

This is a phase I, open label clinical trial that evaluated the effect of *Lactobacillus rhamnosus* GG (LGG), ATCC 53103 on the whole blood transcriptome of elderly subjects. Subjects of 65–80 years of age were recruited from the greater Boston Area using email and hard copy advertisements sent to subjects registered in the Massachusetts General Hospital (MGH) database according to IRB approved protocol (S1 Fig) between December 1, 2010 and August 5, 2011 as previously described [18]. Interested subjects were asked to call the study telephone number, were informed about the study and pre-screened via questionnaire regarding their general good health, whether they consumed yogurt or probiotic on a daily basis, if they were interested in participating in the study and their availability for the required follow-up period. Those interested were scheduled for a screening visit at MGH’s Clinical Research Center (CRC) where subjects completed the consent process, signed the study consent form, gave permission to be tested for HIV, and were asked by study physicians to provide a detailed medical history including current use of medications (prescription and nonprescription), probiotic and dietary supplements. Laboratory tests included complete blood count (CBC), chemistry panel, liver function tests (LFTs), hepatitis B surface antigen, hepatitis C and HIV antibody tests and urine toxicology. At the end of the screening visit, subjects were provided information on foods and probiotic products they should avoid in order to maintain eligibility in the trial. Subjects were contacted by telephone about their eligibility after the lab test results were available, except for those testing positive for HIV, who were asked to return for a follow-up visit at which time the subject was informed of the result, counseled, and referred for further evaluation. Fifteen eligible subjects attended a start up visit where final eligibility criteria were checked and information on the study design, schedule and patient routines and responsibilities were explained prior to the first oral administration of a dose of 1 x 10^10^ colony forming units of LGG per capsule twice daily (1 capsule AM and PM for 28 days) (Fig 1). The LGG capsules were provided by Amerifit Brands Inc., Cromwell, Connecticut and were tested for no evidence of bacteria other than LGG [18]. The first dose was administered under observation at the CRC. Subjects were evaluated during the study at Day 0 (baseline), Day 28 (+/- 2 days), and Day 56 (+/- 1 week), as well as via telephone calls on Days 3 (+/- 1 day),7 (+/- 2 days), 14 (+/- 2days) to record any possible adverse events to the treatment. Compliance with LGG consumption was calculated as the percentage of pills dispensed that were not returned on day 28[18]. Compliance was also estimated based on relative abundance of LGG DNA copies detected in fecal samples of patients throughout the study.

### Clinical sample collection and handling

Venous blood samples were drawn from non-fasted participant (n = 15) at CRC on day 0 (baseline), day 28, and day 56. At each time, blood was collected directly into PAXgene Blood RNA tubes (Preanalytix, Qiagen BD, Valencia, CA) to stabilize blood RNA. After a four hour stabilization period at room temperature, PAXgene tubes with collected blood were frozen at -80°C until further processing. Fecal samples were collected by participants in sterile plastic containers that rested in an H frame that fit into the toilet seat. Subjects were asked to collect samples within 24 hrs of their visits at days 0, 28, and 56 and to place the plastic container with the sample into a styrofoam container surrounded by four ice packs to cool and maintain the specimen at 4°C. Upon arrival, study staff immediately processed the fecal samples into one gram aliquots that were snap frozen at -80°C until further processing. Once all clinical sample collection was completed samples were shipped on dry ice to the USDA/ARS, Beltsville Human Nutrition Research Center, Diet, Genomics and Immunology Laboratory, in Beltsville MD for nucleic acid isolation and processing.

### Isolation of RNA from whole blood samples

RNA was isolated from whole blood using the PAXgene Blood RNA kit from PreAnalytiX [19]. Paxgene tubes were thawed at room temperature for at least three hours. After tubes were centrifuged for 15 min at 4,000 x g the supernatant was discarded and 4 mL of RNAse-free water was added to lyse cells in the pellet. After further centrifugation, pellet matter was treated with different buffers, purified and subjected to on-column DNAse I treatment according to the manufacturer’s instructions. Integrity and quantity of purified RNA was determined via the Experion Automated Electrophoresis Station (Hercules, CA). RNA quality was reported as a score from 1–10 referred to as the RNA Quality Indicator (RQI). RNA samples falling below an RQI threshold of 8.0 were omitted from the study.

### Globin depletion

Following isolation, total RNA samples were depleted of globin mRNA using the GLOBINclear Human Kit as recommended by the manufacturer’s protocol (Ambion, Austin TX)[20]. One microgram of purified RNA was mixed with biotinylated -Globin Capture Oligonucleotides and incubated for 15 min to allow for hybridization. Streptavidin magnetic beads were then used to capture and remove globin mRNA via a magnetic separation. Globin-depleted mRNA was further purified with additional washes using a rapid magnetic bead-based purification method. Quantity and quality of globin-depleted RNA was re-determined using the Experion platform.

### TruSeq Library Prep and Sequencing

The Illumina TruSeq RNA Sample Prep v2 kit (Ilumina, San Diego, USA) was used to prepare the RNA samples for sequencing. Due to limited quantities of high quality RNA available for sequencing, a trial was performed to determine and confirm the minimum quantity of RNA that could be used as input for the TruSeq protocol. RNA inputs of 100, 250, 500 and 1000 ng originating from a single participant were sequenced and gene counts were analyzed for statistical similarity using a matched pair analysis. Conversion of RNA to sequencing libraries involved purifying poly-A containing mRNAs using magnetic beads, fragmenting the molecules, and converting them into cDNA. The cDNA was then subject to end repair, 3’ end adenylation, ligation of Illumina indexing adapters, and PCR enrichment. Libraries were validated for average fragment size and quantified on the Experion Automated Electrophoresis Station using DNA 1K chips. Three libraries were prepared from each subject from samples collected before treatment (Day 0), twenty eight days into daily probiotic consumption (day 28) and after probiotic consumption had been suspended for 28 days (Day 56). Libraries were brought to equimolar concentrations (3–5pM) for cluster generation on Illumina’s cBot prior to being run on the Hi-Seq 2000 sequencer (Illumina,San Diego, CA) for 100 cycles in single-read format.

### Sequence Trimming and Alignment

FASTQ files generated from sequencing were imported into CLC Bio’s Genomics Workbench (v6.5,Aarhus, Denmark). Sequences below a length of 80bp and below a PHRED quality score of 30 were trimmed to ensure 99.9% base call accuracy. Sequences were then aligned to the human reference genome (GRCh37.64) via CLC’s RNA-Seq module with a maximum number of two mismatches, minimum length fraction of 0.95, and a minimum similarity fraction of 0.95, so that at least 95% of bases would map with 95% similarity (http://www.ensembl.org/Homosapiens/Info/Index). Mapped reads for each sample were summarized into gene level expression counts that were used as input for gene expression analysis.

### RNA-Seq Data analysis

Determination of differentially expressed genes (DEG) required an analytical approach tailored to RNA-Seq datasets. For this study we used three statistical tools including Bioconductor packages: edgeR [21], DESeq2 [22], and TSPM. The first two are based on negative binomial generalized linear models (glm) but differ in their normalization and filtering procedures [23]. The third method is based on a two-stage Poisson model (TSPM) [24] that analyzes over-dispersed genes separately from genes that did not exhibit variation significantly greater than the mean (i.e. Poisson distribution). Gene counts representing unique exon reads were chosen for analysis. The time effect was tested using likelihood-ratio statistics to compare data from days 28 and 56 against day 0. By using subject as a blocking variable the time effect was assessed for each patient separately ensuring that baseline differences between subjects were subtracted out. Output from statistical packages included log-fold change (log_2_), log counts per million (or mean by time point), the likelihood ratio statistic (for GLM-based analyses), p-values and FDR-adjusted p-values. Differential expression was determined by fitting a glm using the Cox-Reid profile-adjusted likelihood method for estimating dispersions followed by the likelihood ratio test. P values were corrected using the Benjamini-Hochberg false discovery rate adjustment [25]. In addition, the probability of any specific gene being a false discovery (q-value) was also calculated with the TSPM method [26].DEGs generated from each analysis were compared and used to determine which common genes were differentially expressed. A difference in gene expression was considered significant if the adjusted FDR p-value was < 0.1.

Quality of reads was also checked using a quality control pipeline SolexaQA [27] where nucleotides of each read were scanned for low quality and trimmed. Processed reads were then mapped to the human reference genome using TopHat 2 [28]. SAM output files from TopHat alignment, along with the GTF file from ENSEMBL human genebuild v69.0, were analyzed using Cuffdiff-Cufflink (v1.3.0) to test for differential expression. Mapped reads were normalized based on upper quartile normalization method (-N/—upper-quartile-norm). Cuffdiff models the variance in fragment counts across replicates using the negative binomial distribution as described [29].

### Gene Enrichment

Interpretation of high-throughput gene expression data is facilitated by the consideration of prior biological knowledge [30,31,32,33]. Biological network analysis was performed using Ingenuity Pathway Analysis (IPA) (v 9.0,Ingenuity Systems, Mountain View, CA, USA) to predict potential biological processes, pathways and molecules affected by DEGs. This web-based tool facilitated the association of changes in gene expression with related biological pathways based on a gene’s functional annotation and known molecular interactions. Focus genes were overlaid onto a global molecular network developed from information contained in the IPA Knowledge Base (KB), a large structured collection of observations in various experimental contexts with nearly 5 million findings manually curated and updated from the biomedical literature. The reference network contains ~40,000 nodes that represent mammalian genes and their products, chemical compounds, microRNA molecules and biological functions. Nodes are connected by ~1480000 edges representing experimentally observed cause-effect relationships that relate to expression, transcription, citation, molecular modification, and transport as well as binding events[34]. Networks of these focus genes are algorithmically generated based on their connectivity and number of focus genes. The more focus genes involved, the more likely the association is not due to random chance. In order to identify the networks that are highly expressed, IPA computes a score according to the fit of the genes in the data set. This score is generated using a p-value calculation determined by a right-tailed Fisher’s exact test, and it is displayed as the negative log of that p-value. This score indicates the likelihood that the fit of the focus genes in the network could be explained by chance alone. A score of 2 indicates that there is a 10^−2^ chance that the focus genes are grouped together in a network by chance. A high number of focus genes within a dataset leads to a higher network score. To identify molecules upstream of the affected genes in the dataset, that potentially explained the observed expression changes, the ‘Upstream Regulator Analysis’ (URA) tool within IPA was used. This tool predicted upstream regulators and inferred their activation state by calculating a Z-score to assess the match of observed and predicted up/down regulation patterns. Z-score is particularly suited for pathway analysis since it serves as both a significance measure and a predictor of the activation state of the regulator: activated (Z value >2) or inhibited (Z value <2) [34]. The Downstream Effects Analysis (DEA) was applied and used the methodology of URA for the inference and impact on biological functions and diseases that are down-stream of the genes with altered expression. The goal was to identify those biological processes and functions that were likely to be casually affected by up-and down-regulated genes of our dataset. Graphical presentation of gene-gene interactions and de-regulated genes for enriched pathways are visualized in networks that contain up to 35 genes with an associated score derived from a p- value, indicating the expected likelihood of the genes being present in a network compared to that expected by chance.

To further interpret the biological meaning of DEGs induced in whole blood after *Lactobacillus rhamnosus* consumption for 28 days, we compared the overlap between our gene dataset and Hallmark gene sets from the Molecular Signature Database (MSigDB) [35] so common processes, pathways and underlying biological themes could be identified. The gene sets in the collection that best overlap with the query genes were supported by an FDR adjusted p-value generated from the hypergeometric distribution for the number of genes in the intersection of the query set with a set from MSigDB [35]. To link transcriptome changes induced by probiotic treatment with corresponding patterns produced by human cells in response to biologically active compounds a cross-database analysis using Connectivity–map, C-MAP (build02, http://www.broad.mit.edu/cmap/) was done. The C-MAP is a collection of over 7,000 genome-wide transcriptional expression profiles from cultured human cells treated with over 1300 bioactive small molecules and simple pattern-matching algorithms that together enable the discovery of functional connections between drugs, genes and diseases through the transitory feature of common gene-expression changes[36].

### Fecal DNA RT-PCR analysis

DNA from stool samples provided by participants on days 0, 28, and 56 was isolated using the QIAamp DNA Stool mini-kit (Qiagen, Valencia, CA) [37]. Briefly, 250 mg of a homogenized one- gram fecal sample was weighed and immediately re-suspended with lysis buffer. After heating the suspension at 95°C to increase DNA yield, removal of inhibitors, and proteinase K digestion was done before DNA was bound to a column, washed, and eluted in TE buffer. DNA concentration was determined by the NanoDrop method (Thermo Fisher Scientific, CA). Briefly, 40ng of fecal DNA per sample was used as a template for real time PCR amplification using primers and probes that differentially amplify variable regions within the 16S ribosomal DNA specific for total bacteria [38], *Bifidobacterium species* [39], and *Lactobacillus* species from the *casei* [40] and *non-casei* subgroups [39]. Similarly, relative quantification of LGG abundance was done using a set of primers and probe designed to amplify a highly conserved and ubiquitous *tuf*-gene expressed as a single copy and universally distributed in *Lactobacillus* species [41,42] and used to determine bacterial abundance marker within other probiotic species [37]. The C_T_ values that were generated expressing the target gene’s copy quantity were converted to number of gene copies using standard curves constructed by serially diluting purified fragments of each bacterial gene target. The size of the fragment was verified and molarity was determined by DNA 1K chip using the Experion Automated Electrophoresis System (Biorad, Hercules, CA). A linear relationship was established between the *C*_T_ value and number of target gene copies ranging between10^1^ to 10^10^ copies/mL and this relationship was subsequently used to estimate values of log_10_ target gene copy numbers in fecal samples [43]. All molecular assays were performed on the 7500- Real time PCR System(Perkin Elmer) using a 25 μL PCR amplification mixture containing 1X Thermo-start QPCR master mix with ROX (Abgene, Rochester, NY), forward, reverse, probe and an equivalent of 20 ng of DNA per reaction. The amplification conditions were 50°C for 2 min, 95°C for 10 min, and 40 cycles at 95°C for 15 seconds, and 60°C for 1 min. Mean copy number (expressed as log_10_ target gene copies per gram of feces) was calculated and compared among treatment groups. A one-way repeated measures analysis of variance (ANOVA) model was fit to analyze different bacterial species expressed as copies per gram of feces (cpg) using SAS v9.3 PROC GLIMMIX to specify a lognormal distribution and heterogeneous compound symmetric covariance structure to model correlations among days measured on the same subject and to obtain pair-wise means comparisons among days. Statistical significance among days was reported when p<0.05.

## Results

### LGG treatment compliance and clinical signs

Compliance with LGG based on day 28 (range day 24–day 32) capsule count was 100% in 11 (73%) subjects; between 90–99% in 2 (13%) subjects and 84% in 1 (7%) subjects. Compliance for the final subject could not be estimated because the subject did not return her capsules [18]. LGG treatment compliance was also verified by monitoring changes in *Lactobacillus rhamnosus* abundance in patient fecal samples. A species specific real time PCR assay against a 106 base pair (bp) fragment of the *tuf* gene was designed for identification of *Lactobacillus rhamnosus* species after alignment and comparison with closely related *Lactobacillus* species using the Clustal alignment program [44] (S2 Fig). Forward and reverse primer and probe reagents for LGG detection were tested for specificity using DNA from bacterial reference strains as templates for real time PCR analysis and construction of standard curves as previously described [37]. After 28 days of LGG treatment, there was greater than a three hundred fold increase in LGG copies per gram (cpg) in feces collected (42.05 x 10^5^ ±8.18) when compared to baseline (0.12x 10^5^±0.08) levels or a seven hundred fold increase when compared to day 56 (0.06 x10^5^ ±0.04) levels (P<0.05). Significant differences in LGG copies were not detected between baseline and Day 56. Relative abundance of *Lactobacillus species* from the *casei* group were also significantly increased at day 28 (12.87x 10^5^ ±2.24) when compared to baseline (0.98x 10^5^±0.27) or Day 56 (1.58x 10^5^±0.46)(P<0.05). No other differences were detected in total bacterial counts (Eubacteria), or in *Bifidobacterium species* or *Lactobacillus species* from non-casei group (Table 1). Distribution of blood cell differential data and complete plasma chemistry panels for each participant at baseline (Day 0), day 28 and day 56 were within normal range. No outliers or abnormal patterns were observed at baseline or during LGG feeding (D28 and D56) [18].

**Table 1:** Relative abundance of bacterial species in fecal samples after LGG treatment.

Bacterial species	Collection date			p-value
	0	28	56	
Eubacteria	3.92 x10 ^10^ ± 1.15 ^a^ *	3.82 x10 ^10^ ± 0.68 ^a^	4.80 x10 ^10^ ± 1.14 ^a^	0.2658
*Bifidobacterium spp*.	0.3 x10 ^8^ ± 0.1 ^a^	1.42 x10 ^8^ ± 0.79 ^a^	2.41 x10 ^8^ ± 2.16 ^a^	0.7353
*Lactobacillus spp (non-casei)*	1.55 x10 ^6^ ± 1.13 ^a^	1.55 x10 ^6^ ± 1.36 ^a^	0.48 x10 ^6^ ± 0.22 ^a^	0.256
Lactobacillus spp *(casei)*	0.98 x10 ^5^ ± 0.27 ^a^	12.87 x10 ^5^ ± 2.24 ^**b**^	1.58 x10 ^5^ ± 0.46 ^a^	<0.0001
*Lactobacillus rhamnosus(tuf gene)*	0.12 x10 ^5^ ± 0.08 ^a^	42.05 x10 ^5^ ± 8.18 ^b^	0.06 x10 ^5^ ± 0.04 ^a^	<0.0001

* P-values represent effect of treatment among days.

Any non-identical letters indicate significant difference among collection days (p<0.05).

### Whole blood RNA analysis

Individual gene levels expressed as reads per kilo base per million (RPKM) were compared in a preliminary test among RNA input levels of 100, 250, 500 and 1000ng from a single patient. A matched paired analysis was performed between different RNA input levels and only at 100ng were the count data statistically different from the other input levels (p<0.001). RPKM values were shown to be statistically similar between the 250, 500 and 1000 ng levels, suggesting that a minimum input of 250ng RNA could be used with as much confidence as at the level of 1000 ng (S3 Fig). Based on available RNA quantities, an input of 500 ng was chosen for library preparation and sequencing, if participants had the complete three time point set of high quality RNA (RQI > 8.0) samples. From the fifteen study participants, three samples (401–57 from day 28, 402–28 from day 56 and 409–45 from day 0) were discarded due to low quality, one due to low RNA yield (406–76 day 28) and an additional fourth subject (430–82) was not included in the sequencing analysis due to lack of clinical compliance (S2 Table). Therefore, thirty-two high quality RNA samples from 11 participants were used for the final sequencing analysis (10 participants x 3 time points/subject, 1 participant X 2 time points/subject). Sample randomization of all RNA samples consisted of including an equal number of different time points on each flow cell so as not to repeat the same subject on one flow cell. A mean average of 127.8 ± SD 55.7 million reads per sample was generated. Alignment results showed an average of 76.2±SD 33.7 million unique exon reads from each sample mapped to the human genome similarly to what has been described in other experiments with human blood samples [45] (S3 Table). Reads that uniquely mapped to the reference genome were summarized into gene level expression counts before statistical analysis on platforms edgeR, DESeq2 and TSPM, for the detection of differentially expressed genes.

### Differential Expression of Genes (DEG)

**Fig 2:**
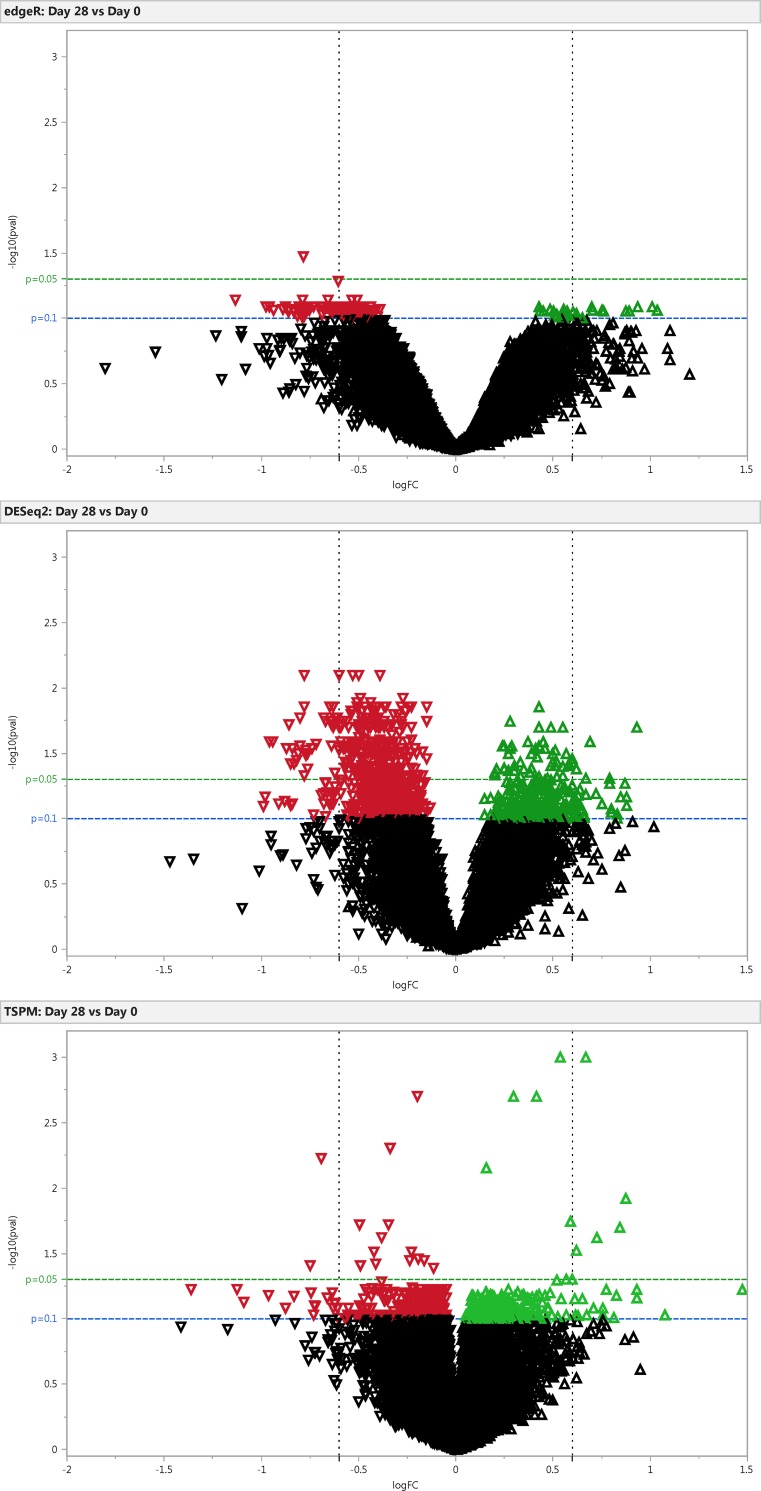
Differential Expression Analysis of RNA-seq Data. Volcano plots depicting the fold difference in gene expression levels after consumption of LGG for 28 days. Volcano plots with DEGs generated from edge-R (Panel A), DESeq2 (Panel B) or TSPM (Panel C) analysis platforms. Colored points in red refer to down-regulated genes green for up-regulated genes according to their fold change (Log FC) in x-axis and p value (log _10_ p-value) p<0.05 or p<0.1 in y-axis.

Our study design had two experimental factors: Subjects (11 levels) and time (three levels per subject). The study was analyzed using a paired sample model in which subjects were used as the blocking factor. Our main goal was to identify genes that were differentially expressed between baseline (day 0) and day 28 after probiotic consumption and between base line and day 56 when probiotic consumption had been suspended for 28 days to see any possible residual probiotic effect. Differential expression analysis was performed on 25,990 annotated genes using the edge-R and DESeq2 Bioconductor packages, the two stage-Poisson model (TSPM), R Script and Cuffdiff analysis tool from Cufflinks. Volcano plots illustrate the general gene expression pattern detected by edgeR, DESeq2 and TSPM using a threshold log fold change of 0.6 (absolute fold change 1.5), with an adjusted FDR p-value<0.05 or <0.1 to capture highly abundant marginal changes in gene expression depending on the analysis platform used (Fig 2). All platforms normalized the count data for library size and removed genes with zero counts across all samples. For edgeR, count data from each gene was run unfiltered (n = 25,990 genes) and also with an inclusion filter of at least 0.1 counts per million (cpm) (n = 13,891 genes), representing a minimum gene count of at least 3 (depending on the library size) in all samples (S4 Table) as suggested in other studies in order to improve statistical power by decreasing the number of multiple comparisons to adjust for and to reduce the possible bias of very small counts with no biological significance [20,46,47,48]. EdgeR-generated DEG using non-filtered data (DEG = 2, FDR p-value<0.1), and with 0.1cpm inclusion filter in all samples (DEG = 139, FDR p-value<0.1) indicated that the gene encoding the low affinity receptor for Fc fragment of Immunoglobulin E (IgE), *FCER2*, was the top common DEG detected in edgeR analyses platforms with a significant 1.7 fold decrease in expression at day 28 (FDR p-value<0.05) (Table 2). Lymphocyte antigen 86 gene, *LY86*, was also down-regulated at day 28 in edge-R analyses with a lower FDR p-value = 0.05 only in 0.1 cpm filtered dataset. An additional group of 137 DEG (111 down, 26 up) with an adjusted FDR p-value<0.1 were only detected in filtered edgeR-dataset (Table 2). DEG were not detected in either edge-R analyses between day 56 and day 0 after LGG consumption had ceased for 28 days (data not shown). The DESeq2 package detected a larger number of DEG (282 down-regulated, 51 up-regulated) changing by at least 1.2 fold with a FDR adjusted p-value<0.05, including *FCER2* and *LY86* among the top four genes with an additional 654 DEG (412 down-regulated, 242 up-regulated) at a higher FDR adjusted p-value threshold of <0.1 (Fig 2) (S5 Table). Similar to edgeR, no DEG were detected with DESeq2 analysis at day 56 when compared to baseline levels (data not shown). Genes that met the count abundance criteria with mean counts of at least 1 in a minimum of 2 samples with non-zero counts (n = 19,575) were used for TSPM analysis. A total of 890 and 63 DEGs were identified with an over-dispersed and Poisson gene distribution, respectively. At day 28 -, 953 DEG (574 down-regulated, 379 up-regulated) with adjusted FDR p-value<0.1 were identified, only 29 with a FDR-adjusted p-value <0.05 (S6 Table), however, most of the changes were less than the 0.6 log fold cutoff (Fig 2). At day 56, only a few DEG with Poisson distribution were detected (adjusted FDR p-value <0.1, log fold <0.6) (data not shown). When edgeR, DESeq2 and TSPM DEG lists were compared 95 common DEG (77 down-regulated, 18 up-regulated) (FDR p-value <0.1) were identified across all three analysis platforms (S4 Fig). Several DEG (n = 19) with very low cpm were detected by edgeR and TSPM but not by DESeq2 (i.e, *RNASE1*, *SIGLEC11*,*C1orf132*, *ZNF593*, *SFTPD*, *CBLN3*, *SLC35E2*,*GLIS3*, *PXMP2*, *C10orf98*, *FUT10*, *COCH*, *ESM1*, *LYPD2*, *CLEC11A*, *LIPC*, *SYCE1L*, *LBRC24*,*PLEKHM3)* (Table 2). Cuffdiff differential expression analysis also detected similar fold changes as DESeq2 for common DEG; however none reached statistical significance (data not shown).

**Table 2:** Common whole blood DEG identified by different RNA-seq analysis platform in elderly subjects after a 28 day treatment with LGG.

						Analysis platform											
Symbol	Gene ID	Locus	Description	Location	Regulation direction	edgeR-NF			edgeR cpm0.1/all			DESeq2			TSPM		
						logFC	FC	padj	logFC	FC	padj	logFC	FC	padj	logFC	FC	padj
FCER2	ENSG00000104921	19:7753643–7767032	Fc fragment of IgE, low affinity II, receptor for (CD23)	Plasma Membrane	down	-0.77	1.71	0.047 *	-0.78	1.72	0.034 *	-0.78	1.72	0.008 *	-0.26	1.20	0.063
LY86	ENSG00000112799	6:6346697–6655216	lymphocyte antigen 86	Plasma Membrane	down	-0.59	1.51	0.084	-0.60	1.52	0.052 *	-0.60	1.52	0.008 *	-0.18	1.13	0.067
DYNLL1	ENSG00000088986	12:120907652–120936296	dynein, light chain, LC8-type 1	Cytoplasm	down	-0.52	1.44	0.137	-0.53	1.45	0.073	-0.53	1.44	0.008 *	-0.18	1.13	0.060
CD79B	ENSG00000007312	17:62006099–62009714	CD79b molecule, immunoglobulin-associated beta	Plasma Membrane	down	-0.50	1.41	0.137	-0.51	1.42	0.073	-0.50	1.41	0.008 *	-0.18	1.13	0.073
VPREB3	ENSG00000128218	22:24094929–24096655	pre-B lymphocyte 3	Cytoplasm	down	-0.78	1.71	0.137	-0.79	1.73	0.073	-0.78	1.72	0.014 *	-0.25	1.19	0.081
LGALS1	ENSG00000100097	22:38071614–38075813	lectin, galactoside-binding, soluble, 1	Extracellular Space	down	-0.65	1.56	0.137	-0.66	1.58	0.073	-0.65	1.57	0.014 *	-0.22	1.16	0.081
*RNASE1*	ENSG00000129538	14:21269386–21271437	ribonuclease, RNase A family, 1 (pancreatic)	Extracellular Space	down	-1.12	2.17	0.137	-1.13	2.19	0.073	-1.10	2.14	NA	-0.37	1.29	0.065
HLA-DRB1	ENSG00000206306	6:32546545–32557625	major histocompatibility complex, class II, DR beta 1	Plasma Membrane	down	-0.48	1.39	0.140	-0.49	1.40	0.082	-0.49	1.40	0.012 *	-0.18	1.13	0.060
ATP6V1F	ENSG00000128524	7:128470430–128550773	ATPase, H+ transporting, lysosomal 14kDa, V1 subunit F	Other	down	-0.50	1.42	0.145	-0.51	1.43	0.082	-0.51	1.42	0.013 *	-0.17	1.13	0.065
HLA-DMB	ENSG00000241674	HSCHR6_MHC_DBB:32880345–32898843	major histocompatibility complex, class II, DM beta	Plasma Membrane	down	-0.43	1.34	0.145	-0.44	1.36	0.082	-0.44	1.36	0.013 *	-0.13	1.10	0.080
LSMD1	ENSG00000183011	17:7760002–7816078	LSM domain containing 1	Other	down	-0.62	1.54	0.137	-0.64	1.55	0.082	-0.63	1.55	0.014 *	-0.19	1.14	0.084
PTRHD1	ENSG00000184924	2:25012854–25142708	peptidyl-tRNA hydrolase domain containing 1	Other	down	-0.63	1.54	0.145	-0.64	1.56	0.082	-0.63	1.55	0.014 *	-0.19	1.14	0.089
TIMM13	ENSG00000099800	19:2389768–2456994	translocase of inner mitochondrial membrane 13 homolog (yeast)	Cytoplasm	down	-0.50	1.42	0.145	-0.51	1.43	0.082	-0.51	1.42	0.014 *	-0.18	1.13	0.065
AKR1A1	ENSG00000117448	1:46016214–46035721	aldo-keto reductase family 1, member A1 (aldehyde reductase)	Cytoplasm	down	-0.46	1.38	0.145	-0.47	1.39	0.082	-0.47	1.39	0.014 *	-0.15	1.11	0.069
TPR	ENSG00000047410	1:186265404–186344825	translocated promoter region, nuclear basket protein	Nucleus	up	0.44	1.36	0.145	0.43	1.35	0.082	0.43	1.35	0.014 *	0.15	1.11	0.063
GSTO1	ENSG00000148834	10:105995113–106027217	glutathione S-transferase omega 1	Cytoplasm	down	-0.52	1.44	0.145	-0.53	1.45	0.082	-0.53	1.44	0.015 *	-0.16	1.12	0.088
CAPG	ENSG00000042493	2:85621345–85645555	capping protein (actin filament), gelsolin-like	Nucleus	down	-0.48	1.39	0.145	-0.49	1.40	0.082	-0.48	1.39	0.015 *	-0.16	1.12	0.065
MRPL11	ENSG00000174547	11:66202545–66234209	mitochondrial ribosomal protein L11	Cytoplasm	down	-0.53	1.45	0.145	-0.54	1.46	0.082	-0.54	1.45	0.016 *	-0.17	1.12	0.083
CFD	ENSG00000197766	19:859452–863569	complement factor D (adipsin)	Extracellular Space	down	-0.80	1.74	0.137	-0.81	1.75	0.082	-0.80	1.74	0.017 *	-0.36	1.28	0.060
POLE4	ENSG00000115350	2:75185618–75197255	polymerase (DNA-directed), epsilon 4, accessory subunit	Nucleus	down	-0.67	1.59	0.145	-0.69	1.61	0.082	-0.68	1.60	0.017 *	-0.20	1.15	0.102
FAU	ENSG00000149806	11:64888099–64902004	Finkel-Biskis-Reilly murine sarcoma virus (FBR-MuSV) ubiquitously expressed	Cytoplasm	down	-0.61	1.53	0.145	-0.62	1.54	0.082	-0.62	1.54	0.017 *	-0.19	1.14	0.093
IFI27L2	ENSG00000119632	14:94594115–94596590	interferon, alpha-inducible protein 27-like 2	Other	down	-0.67	1.59	0.145	-0.68	1.60	0.082	-0.67	1.59	0.018 *	-0.21	1.16	0.093
UQCR11	ENSG00000267059	19:1576676–1605483	ubiquinol-cytochrome c reductase, complex III subunit XI	Cytoplasm	down	-0.64	1.56	0.145	-0.65	1.57	0.082	-0.64	1.56	0.018 *	-0.20	1.14	0.094
NAGK	ENSG00000124357	2:71163011–71306935	N-acetylglucosamine kinase	Cytoplasm	down	-0.57	1.48	0.145	-0.58	1.50	0.082	-0.58	1.49	0.018 *	-0.16	1.12	0.093
ROMO1	ENSG00000125995	20:34213952–34288906	reactive oxygen species modulator 1	Cytoplasm	down	-0.63	1.55	0.145	-0.64	1.56	0.082	-0.64	1.56	0.019 *	-0.21	1.15	0.083
PRDX4	ENSG00000123131	X:23682378–23704516	peroxiredoxin 4	Cytoplasm	down	-0.59	1.50	0.145	-0.60	1.52	0.082	-0.59	1.51	0.019 *	-0.19	1.14	0.093
CD52	ENSG00000169442	1:26605666–26647014	CD52 molecule	Plasma Membrane	down	-0.86	1.82	0.145	-0.88	1.84	0.082	-0.86	1.82	0.019 *	-0.25	1.19	0.123
ERN1	ENSG00000178607	17:62120352–62207504	endoplasmic reticulum to nucleus signaling 1	Cytoplasm	up	0.95	1.93	0.145	0.94	1.91	0.082	0.93	1.91	0.02 *	0.34	1.27	0.066
ATP5H	ENSG00000167863	17:73028669–73061984	ATP synthase, H+ transporting, mitochondrial Fo complex, subunit d	Other	down	-0.59	1.50	0.145	-0.60	1.52	0.082	-0.59	1.51	0.02 *	-0.18	1.14	0.093
VAMP8	ENSG00000118640	2:85788684–85809154	vesicle-associated membrane protein 8	Plasma Membrane	down	-0.59	1.51	0.145	-0.61	1.52	0.082	-0.60	1.52	0.02 *	-0.18	1.13	0.103
RNASE6	ENSG00000169413	14:21249209–21250626	ribonuclease, RNase A family, k6	Extracellular Space	down	-0.63	1.54	0.145	-0.64	1.56	0.082	-0.63	1.55	0.02 *	-0.21	1.16	0.083
NBPF14	ENSG00000122497	1:148003641–148025863	neuroblastoma breakpoint family, member 15	Other	up	0.71	1.64	0.145	0.70	1.62	0.082	0.69	1.61	0.026 *	0.25	1.19	0.081
RPS15A	ENSG00000134419	16:18792616–18813000	ribosomal protein S15a	Cytoplasm	down	-0.96	1.95	0.145	-0.98	1.97	0.082	-0.96	1.95	0.026 *	-0.28	1.22	0.145
RPL35	ENSG00000136942	9:127615754–127624260	ribosomal protein L35	Cytoplasm	down	-0.72	1.64	0.145	-0.73	1.66	0.082	-0.72	1.65	0.027 *	-0.22	1.16	0.118
*SIGLEC11*	ENSG00000161640	19:50392910–50464429	sialic acid binding Ig-like lectin 11	Plasma Membrane	down	-0.95	1.93	0.145	-0.96	1.94	0.082	-0.95	1.93	NA	-0.36	1.29	0.072
*C1orf132*	ENSG00000203709	1:207986904–208042495	Chromosome 1 open reading frame 32		up	1.02	2.03	0.145	1.01	2.01	0.082	1.00	2.00	NA	0.42	1.34	0.067
*ZNF593*	ENSG00000142684	1:26496361–26498551	zinc finger protein 593	Nucleus	down	-0.69	1.61	0.145	-0.70	1.62	0.082	-0.69	1.61	NA	-0.26	1.20	0.066
*SFTPD*	ENSG00000133661	10:81664653–81742370	surfactant protein D	Extracellular Space	down	-0.76	1.69	0.145	-0.76	1.69	0.082	-0.75	1.68	NA	-0.22	1.16	0.083
ATP5G3	ENSG00000154518	2:176040985–176049335	ATP synthase, H+ transporting, mitochondrial Fo complex, subunit C3 (subunit 9)	Cytoplasm	down	-0.38	1.30	0.147	-0.39	1.31	0.085	-0.39	1.31	0.008 *	-0.13	1.09	0.060
C11orf31	ENSG00000211450	11:57480071–57587018	chromosome 11 open reading frame 31	Nucleus	down	-0.55	1.47	0.145	-0.57	1.48	0.085	-0.56	1.47	0.018 *	-0.19	1.14	0.083
CHCHD2	ENSG00000106153	7:56169261–56174269	coiled-coil-helix-coiled-coil-helix domain containing 2	Cytoplasm	down	-0.47	1.38	0.145	-0.48	1.39	0.085	-0.48	1.39	0.018 *	-0.15	1.11	0.083
FLVCR1	ENSG00000162769	1:213031596–213072705	feline leukemia virus subgroup C cellular receptor 1	Plasma Membrane	up	0.57	1.48	0.145	0.55	1.47	0.085	0.55	1.46	0.02 *	0.24	1.18	0.065
LILRB4	ENSG00000186818	19:55155339–55181810	leukocyte immunoglobulin-like receptor, subfamily B (with TM and ITIM domains), member 4	Plasma Membrane	down	-0.54	1.46	0.145	-0.55	1.47	0.085	-0.55	1.46	0.02 *	-0.18	1.13	0.082
RPL15	ENSG00000174748	3:23933150–24021237	ribosomal protein L15	Cytoplasm	down	-0.49	1.40	0.145	-0.50	1.41	0.085	-0.50	1.41	0.02 *	-0.16	1.12	0.083
PRRC2C	ENSG00000117523	1:171454650–171562650	proline-rich coiled-coil 2C	Cytoplasm	up	0.50	1.41	0.145	0.49	1.40	0.085	0.49	1.40	0.02 *	0.16	1.12	0.067
CHI3L2	ENSG00000064886	1:111729795–111786062	chitinase 3-like 2	Extracellular Space	down	-0.80	1.74	0.145	-0.81	1.76	0.085	-0.80	1.74	0.028 *	-0.37	1.29	0.072
NDUFA4	ENSG00000065518	3:120315155–120321347	NADH dehydrogenase (ubiquinone) 1 alpha subcomplex, 4, 9kDa	Cytoplasm	down	-0.87	1.83	0.145	-0.89	1.85	0.085	-0.87	1.83	0.029 *	-0.25	1.19	0.143
RPL27	ENSG00000131469	17:41150289–41154976	ribosomal protein L27	Cytoplasm	down	-0.84	1.79	0.145	-0.86	1.81	0.085	-0.84	1.79	0.029 *	-0.25	1.19	0.147
RPS21	ENSG00000171858	1:150266288–150281414	ribosomal protein S21	Cytoplasm	down	-0.76	1.70	0.145	-0.78	1.71	0.085	-0.77	1.71	0.03 *	-0.23	1.17	0.135
*CBLN3*	ENSG00000139899	14:24895737–24912111	cerebellin 3 precursor	Extracellular Space	down	-0.63	1.55	0.145	-0.64	1.56	0.085	-0.63	1.55	NA	-0.17	1.12	0.093
CCDC106	ENSG00000173581	19:56146381–56164527	coiled-coil domain containing 106	Extracellular Space	down	-0.61	1.53	0.145	-0.62	1.54	0.086	-0.61	1.53	0.017 *	-0.22	1.17	0.065
C12orf57	ENSG00000111678	12:7052140–7055166	chromosome 12 open reading frame 57	Other	down	-0.58	1.50	0.145	-0.59	1.51	0.086	-0.59	1.51	0.026 *	-0.20	1.15	0.087
BRK1	ENSG00000254999	3:10157275–10168874	BRICK1, SCAR/WAVE actin-nucleating complex subunit	Cytoplasm	down	-0.46	1.38	0.145	-0.48	1.39	0.086	-0.47	1.39	0.019 *	-0.14	1.10	0.093
RNASE3	ENSG00000064886	14:21359557–21360507	ribonuclease, RNase A family, 3	Extracellular Space	down	-0.92	1.89	0.145	-0.94	1.92	0.087	-0.94	1.92	0.026 *	-0.28	1.21	0.093
C17orf79	ENSG00000172301	17:30178882–30186356	coordinator of PRMT5, differentiation stimulator	Nucleus	down	-0.50	1.42	0.145	-0.51	1.43	0.087	-0.51	1.42	0.014 *	-0.18	1.13	0.063
PRADC1	ENSG00000135617	2:73455133–73460366	protease-associated domain containing 1	Extracellular Space	down	-0.50	1.41	0.145	-0.51	1.42	0.087	-0.50	1.41	0.014 *	-0.18	1.13	0.065
C10orf125		10:98741040–98745582	fucose mutarotase	Other	down	-0.53	1.44	0.145	-0.54	1.45	0.087	-0.54	1.45	0.015 *	-0.18	1.13	0.074
FAM96B	ENSG00000166595	16:66965958–66968326	family with sequence similarity 96, member B	Cytoplasm	down	-0.46	1.38	0.145	-0.48	1.39	0.087	-0.47	1.39	0.018 *	-0.16	1.12	0.077
HLA-DMA	ENSG00000204257	6:32902405–32949282	major histocompatibility complex, class II, DM alpha	Plasma Membrane	down	-0.44	1.36	0.145	-0.45	1.37	0.087	-0.45	1.37	0.018 *	-0.15	1.11	0.081
MT-ND3	ENSG00000198840	MT:10058–10404	NADH dehydrogenase, subunit 3 (complex I)	Cytoplasm	down	-0.55	1.46	0.145	-0.56	1.47	0.087	-0.55	1.46	0.02 *	-0.21	1.16	0.066
LSM7	ENSG00000130332	19:2321519–2328615	LSM7 homolog, U6 small nuclear RNA associated (S. cerevisiae)	Nucleus	down	-0.64	1.55	0.145	-0.65	1.57	0.087	-0.64	1.56	0.028 *	-0.20	1.15	0.100
COX4I1	ENSG00000131143	16:85805363–85840650	cytochrome c oxidase subunit IV isoform 1	Cytoplasm	down	-0.56	1.48	0.145	-0.58	1.49	0.087	-0.57	1.48	0.028 *	-0.17	1.13	0.102
UBL5	ENSG00000198258	19:9938567–9940791	ubiquitin-like 5	Cytoplasm	down	-0.57	1.48	0.145	-0.58	1.50	0.087	-0.57	1.48	0.029 *	-0.16	1.12	0.144
RPS18	ENSG00000096150	HSCHR6_MHC_QBL:33129804–33173129	ribosomal protein S18	Cytoplasm	down	-0.74	1.67	0.145	-0.75	1.68	0.087	-0.74	1.67	0.029 *	-0.22	1.16	0.151
COX7A2	ENSG00000112695	6:75947390–75960039	cytochrome c oxidase subunit VIIa polypeptide 2 (liver)	Cytoplasm	down	-0.59	1.51	0.148	-0.61	1.52	0.087	-0.60	1.52	0.03 *	-0.17	1.12	0.147
RPL35A	ENSG00000182899	3:197615945–197687013	ribosomal protein L35a	Cytoplasm	down	-0.75	1.69	0.145	-0.77	1.70	0.087	-0.76	1.69	0.032 *	-0.24	1.18	0.138
TCL1A	ENSG00000100721	14:96176303–96223993	T-cell leukemia/lymphoma 1A	Nucleus	down	-0.76	1.70	0.145	-0.77	1.71	0.087	-0.77	1.71	0.033 *	-0.28	1.21	0.106
*SLC35E2*	ENSG00000189339	1:1634168–1677431	solute carrier family 35, member E2	Other	up	0.71	1.64	0.145	0.70	1.63	0.087	0.70	1.62	NA	0.24	1.18	0.081
*GLIS3*	ENSG00000107249	9:3824126–4348392	GLIS family zinc finger 3	Nucleus	up	1.05	2.06	0.156	1.04	2.05	0.087	1.02	2.03	NA	0.41	1.33	0.083
*PXMP2*	ENSG00000176894	12:133200344–133532892	peroxisomal membrane protein 2, 22kDa	Cytoplasm	down	-0.63	1.55	0.159	-0.64	1.56	0.087	-0.63	1.55	NA	-0.19	1.14	0.093
*C1orf98*	ENSG00000203721	1:200311671–200343482	long intergenic non-protein coding RNA 862	Other	up	0.76	1.70	0.147	0.75	1.69	0.087	0.75	1.68	NA	0.24	1.18	0.083
COMMD9	ENSG00000110442	11:36295050–36310999	COMM domain containing 9	Other	down	-0.40	1.32	0.156	-0.41	1.33	0.088	-0.41	1.33	0.015 *	-0.13	1.09	0.073
MPDU1	ENSG00000129255	17:7465191–7536700	mannose-P-dolichol utilization defect 1	Cytoplasm	down	-0.41	1.33	0.156	-0.42	1.34	0.088	-0.42	1.34	0.016 *	-0.14	1.10	0.066
NDUFB9	ENSG00000147684	8:125500725–125740730	NADH dehydrogenase (ubiquinone) 1 beta subcomplex, 9, 22kDa	Cytoplasm	down	-0.43	1.34	0.145	-0.44	1.35	0.088	-0.43	1.35	0.017 *	-0.13	1.10	0.081
RNASEH2C	ENSG00000172922	11:65479466–65488418	ribonuclease H2, subunit C	Other	down	-0.45	1.37	0.145	-0.46	1.38	0.088	-0.46	1.38	0.017 *	-0.16	1.12	0.067
EIF4EBP1	ENSG00000187840	8:37887858–37917883	eukaryotic translation initiation factor 4E binding protein 1	Cytoplasm	down	-0.49	1.41	0.145	-0.50	1.42	0.088	-0.50	1.41	0.019 *	-0.17	1.13	0.072
TRERF1	ENSG00000124496	6:42192668–42419789	transcriptional regulating factor 1	Nucleus	up	0.44	1.36	0.151	0.43	1.35	0.088	0.43	1.35	0.02 *	0.16	1.12	0.072
ASGR1	ENSG00000141505	17:7076749–7082883	asialoglycoprotein receptor 1	Plasma Membrane	down	-0.51	1.42	0.152	-0.52	1.43	0.088	-0.51	1.42	0.02 *	-0.17	1.13	0.082
SF3B5	ENSG00000169976	6:144416017–144416754	splicing factor 3b, subunit 5, 10kDa	Nucleus	down	-0.46	1.38	0.149	-0.47	1.39	0.088	-0.47	1.39	0.025 *	-0.15	1.11	0.083
SERF2	ENSG00000140264	15:44019115–44095241	small EDRK-rich factor 2	other	down	-0.50	1.42	0.145	-0.51	1.43	0.088	-0.51	1.42	0.026 *	-0.18	1.13	0.093
GADD45GIP1	ENSG00000179271	9:92219927–92221470	growth arrest and DNA-damage-inducible, gamma interacting protein 1	Nucleus	down	-0.54	1.45	0.145	-0.55	1.46	0.088	-0.54	1.45	0.026 *	-0.19	1.14	0.083
GPX4	ENSG00000167468	19:1103935–1106787	glutathione peroxidase 4	Cytoplasm	down	-0.48	1.40	0.145	-0.49	1.41	0.088	-0.49	1.40	0.026 *	-0.17	1.13	0.077
TMEM141	ENSG00000244187	7:134671258–134855547	transmembrane protein 141	Other	down	-0.52	1.43	0.147	-0.53	1.44	0.088	-0.52	1.43	0.026 *	-0.18	1.13	0.081
UPF2	ENSG00000151461	10:11962020–12085169	UPF2 regulator of nonsense transcripts homolog (yeast)	Cytoplasm	up	0.46	1.38	0.156	0.45	1.36	0.088	0.45	1.37	0.026 *	0.17	1.12	0.069
C19orf53	ENSG00000104979	19:13875345–13889276	chromosome 19 open reading frame 53	Other	down	-0.51	1.42	0.145	-0.52	1.43	0.088	-0.51	1.42	0.028 *	-0.18	1.13	0.083
C6orf108	ENSG00000112667	6:43193366–43197222	2'-deoxynucleoside 5'-phosphate N-hydrolase 1	Nucleus	down	-0.59	1.50	0.156	-0.60	1.52	0.088	-0.59	1.51	0.028 *	-0.20	1.15	0.093
UQCR10	ENSG00000184076	22:30163357–30166402	ubiquinol-cytochrome c reductase, complex III subunit X	Cytoplasm	down	-0.51	1.42	0.152	-0.52	1.44	0.088	-0.52	1.43	0.029 *	-0.16	1.12	0.093
RNASE2	ENSG00000169385	14:21423610–21424595	ribonuclease, RNase A family, 2 (liver, eosinophil-derived neurotoxin)	Cytoplasm	down	-0.79	1.73	0.156	-0.81	1.75	0.088	-0.81	1.75	0.032 *	-0.27	1.21	0.083
MLL2	ENSG00000167548	12:49388931–49453557			up	0.51	1.43	0.156	0.50	1.42	0.088	0.50	1.41	0.032 *	0.19	1.14	0.083
IL18R1	ENSG00000115604	2:102927961–103015218	interleukin 18 receptor 1	Plasma Membrane	up	0.59	1.50	0.152	0.57	1.49	0.088	0.57	1.48	0.032 *	0.24	1.18	0.069
COX7C	ENSG00000127184	5:85913720–85916779	cytochrome c oxidase subunit VIIc	Cytoplasm	down	-0.84	1.79	0.156	-0.86	1.82	0.088	-0.85	1.80	0.038 *	-0.26	1.19	0.164
IFITM3	ENSG00000142089	11:319668–321340	interferon induced transmembrane protein 3	Plasma Membrane	down	-0.85	1.80	0.156	-0.86	1.82	0.088	-0.83	1.78	0.038 *	-0.24	1.18	0.102
*FUT10*	ENSG00000172728	8:33228341–33371119	fucosyltransferase 10 (alpha (1,3) fucosyltransferase)	Cytoplasm	up	0.88	1.84	0.147	0.87	1.83	0.088	0.86	1.82	NA	0.37	1.29	0.069
*COCH*	ENSG00000100473	14:31343719–31562818	cochlin	Extracellular Space	up	0.77	1.71	0.156	0.76	1.70	0.088	0.75	1.68	NA	0.22	1.16	0.098
*ESM1*	ENSG00000164283	5:54273691–54330398	endothelial cell-specific molecule 1	Extracellular Space	up	0.90	1.87	0.163	0.89	1.85	0.088	0.88	1.84	NA	0.34	1.26	0.083
MZT2B	ENSG00000152082	2:130908980–130948302	mitotic spindle organizing protein 2B	Cytoplasm	down	-0.55	1.46	0.148	-0.55	1.47	0.089	-0.55	1.46	0.029 *	-0.20	1.15	0.079
MRPL23	ENSG00000214026	11:1968507–2011150	mitochondrial ribosomal protein L23	Cytoplasm	down	-0.44	1.36	0.156	-0.45	1.37	0.089	-0.45	1.37	0.018 *	-0.14	1.10	0.083
SYNE2	ENSG00000054654	14:64319682–64805317	spectrin repeat containing, nuclear envelope 2	Nucleus	up	0.62	1.53	0.159	0.60	1.52	0.089	0.60	1.52	0.037 *	0.22	1.17	0.093
*LYPD2*	ENSG00000197353	8:143831567–143833952	LY6/PLAUR domain containing 2	Other	down	-0.62	1.54	0.161	-0.63	1.55	0.089	-0.63	1.55	NA	-0.19	1.14	0.083
RBX1	ENSG00000100387	22:41253080–41369313	ring-box 1, E3 ubiquitin protein ligase	Cytoplasm	down	-0.81	1.76	0.164	-0.83	1.78	0.089	-0.82	1.77	0.036 *	-0.25	1.19	0.129
RPSA	ENSG00000168028	3:39448179–39453929	ribosomal protein SA	Cytoplasm	down	-0.50	1.42	0.156	-0.52	1.43	0.090	-0.51	1.42	0.029 *	-0.17	1.13	0.094
RPS9	ENSG00000170889	19:54704609–54752862	ribosomal protein S9	Cytoplasm	down	-0.58	1.49	0.145	-0.59	1.51	0.091	-0.58	1.49	0.029 *	-0.20	1.15	0.093
RPS5	ENSG00000083845	2:95752951–95831158	ribosomal protein S5	Other	down	-0.53	1.44	0.145	-0.54	1.45	0.091	-0.53	1.44	0.028 *	-0.18	1.13	0.089
RAB34	ENSG00000109113	17:27041298–27045447	RAB34, member RAS oncogene family	Cytoplasm	down	-0.42	1.34	0.159	-0.43	1.35	0.092	-0.43	1.35	0.015 *	-0.14	1.10	0.077
BLOC1S1	ENSG00000135441	12:56075329–56118489	biogenesis of lysosomal organelles complex-1, subunit 1	Cytoplasm	down	-0.52	1.44	0.156	-0.54	1.45	0.092	-0.53	1.44	0.026 *	-0.15	1.11	0.100
RPLP0	ENSG00000089157	12:120634488–120639038	ribosomal protein, large, P0	Cytoplasm	down	-0.49	1.40	0.145	-0.50	1.41	0.092	-0.50	1.41	0.024 *	-0.16	1.12	0.093
BOD1L	ENSG00000038219	4:13570361–13629347	biorientation of chromosomes in cell division 1-like 1	Extracellular Space	up	0.64	1.55	0.166	0.62	1.54	0.092	0.62	1.54	0.042 *	0.23	1.17	0.083
PNOC	ENSG00000168081	8:28107579–28200872	prepronociceptin	Extracelular space	down	-0.49	1.40	0.171	-0.50	1.41	0.094	-0.50	1.41	0.014 *	-0.17	1.13	0.069
NCOA1	ENSG00000084676	2:24714782–24993571	nuclear receptor coactivator 1	Nucleus	up	0.53	1.44	0.164	0.52	1.43	0.094	0.52	1.43	0.037 *	0.19	1.14	0.083
ZCCHC6	ENSG00000083223	9:88902647–88969369	zinc finger, CCHC domain containing 6	Other	up	0.57	1.48	0.166	0.56	1.47	0.094	0.55	1.46	0.04 *	0.21	1.16	0.083
LGALS2	ENSG00000100079	22:37966254–37978623	lectin, galactoside-binding, soluble, 2	Cytoplasm	down	-0.76	1.69	0.166	-0.78	1.71	0.094	-0.76	1.69	0.042 *	-0.16	1.12	0.207
*CLEC11A*	ENSG00000105472	19:51226585–51228974	C-type lectin domain family 11, member A	Extracellular Space	down	-0.68	1.60	0.163	-0.69	1.61	0.094	-0.68	1.60	NA	-0.32	1.25	0.065
VAMP5	ENSG00000168899	2:85811530–85820535	Vesicle associated membrane protein	Plasma Membrane	down	-0.53	1.44	0.166	-0.54	1.45	0.095	-0.53	1.44	0.034 *	-0.17	1.13	0.094
BCL2L11	ENSG00000153094	2:111876954–111924587	BCL2-like 11 (apoptosis facilitator)	Cytoplasm	up	0.59	1.51	0.166	0.58	1.50	0.095	0.58	1.49	0.039 *	0.21	1.16	0.088
ECI1	ENSG00000167969	16:2289395–2302301	Enoyl-CoA delta isomerase 1	Cytoplasm	down	-0.45	1.36	0.161	-0.46	1.37	0.095	-0.45	1.37	0.02 *	-0.15	1.11	0.074
MGMT	ENSG00000170430	10:131265447–131566271	O-6 methylguanine-DNA methyl-transferase	Nucleus	down	-0.48	1.39	0.156	-0.49	1.40	0.095	-0.48	1.39	0.021 *	-0.17	1.12	0.067
C11orf51			anaphase promoting complex subunit 15	Other	down	-0.52	1.44	0.164	-0.54	1.45	0.095	-0.53	1.44	0.028 *	-0.15	1.11	0.121
TPPP3	ENSG00000159713	16:67423711–67427438	tubulin polymerization-promoting protein family member 3	Other	down	-0.53	1.44	0.156	-0.54	1.45	0.095	-0.53	1.44	0.03 *	-0.16	1.12	0.110
MFN2	ENSG00000116688	1:12040237–12073571	mitofusin 2	Cytoplasm	up	0.51	1.43	0.157	0.51	1.42	0.095	0.50	1.41	0.037 *	0.19	1.14	0.088
SIPA1L2	ENSG00000116991	1:232533710–232697304	signal-induced proliferation-associated 1 like 2	Other	up	0.59	1.50	0.159	0.58	1.49	0.095	0.58	1.49	0.038 *	0.21	1.15	0.083
KAT6A	ENSG00000083168	8:41786996–41909508	K(lysine) acetyltransferase 6A	Nucleus	up	0.52	1.43	0.166	0.51	1.42	0.095	0.51	1.42	0.039 *	0.18	1.13	0.083
HLA-DRA	ENSG00000204287	6:32407618–32412823	Major Histocompatibility complex class II, DR alpha	Plasma Membrane	down	-0.47	1.38	0.145	-0.48	1.39	0.096	-0.48	1.39	0.017 *	-0.15	1.11	0.085
C7orf50	ENSG00000146540	7:1036622–1177896	chromosome 7 open reading frame 50	Other	down	-0.47	1.39	0.159	-0.48	1.39	0.096	-0.47	1.39	0.029 *	-0.17	1.12	0.083
SSR4	ENSG00000180879	X:153051220–153063960	Signal sequence receptor delta	Cytoplasm	down	-0.47	1.39	0.161	-0.48	1.40	0.096	-0.48	1.39	0.032 *	-0.16	1.12	0.083
NDUFB7	ENSG00000099795	19:14676889–14682874	NADH dehydrogenase (ubiquinone) 1 beta subcomplex, 7, 18kDa	Cytoplasm	down	-0.56	1.48	0.161	-0.57	1.49	0.096	-0.56	1.47	0.037 *	-0.20	1.15	0.093
*LIPC*	ENSG00000166035	15:58245621–58861151	Hepatic lipase	Extracellular Space	down	-0.77	1.71	0.181	-0.78	1.72	0.096	-0.77	1.71	NA	-0.22	1.16	0.123
*SYCE1L*	ENSG00000205078	16:77224731–77478233	Synaptonemal complex central element protein 2	Other	down	-0.81	1.75	0.181	-0.81	1.76	0.096	-0.80	1.74	NA	-0.27	1.20	0.097
RPS13	ENSG00000110700	11:17095935–17229530	Ribosomal protein S3	Other	down	-0.54	1.45	0.156	-0.55	1.46	0.097	-0.54	1.45	0.036 *	-0.17	1.13	0.110
CSTB	ENSG00000160213	21:45192392–45196326	cystatin B (stefin B)	Cytoplasm	down	-0.39	1.31	0.166	-0.40	1.32	0.098	-0.40	1.32	0.017 *	-0.13	1.10	0.063
S100A6	ENSG00000197956	1:153506078–153508720	S100 Calcium binding protein A6	Cytoplasm	down	-0.46	1.37	0.145	-0.47	1.38	0.098	-0.46	1.38	0.022 *	-0.14	1.10	0.093
C11orf73	ENSG00000149196	11:86013252–86056969	Chromosome 11 open reading frame 73	cytoplasm	down	-0.45	1.37	0.181	-0.47	1.38	0.098	-0.46	1.38	0.026 *	-0.14	1.10	0.093
MFSD9	ENSG00000135953	2:103332298–103353347	major facilitator superfamily domain containing 9	Other	up	0.53	1.44	0.161	0.52	1.43	0.098	0.51	1.42	0.03 *	0.20	1.15	0.082
*LRRC24*	ENSG00000254402	8:145743375–145754516	Leucine rich repeat containing 24	other	down	-0.66	1.57	0.181	-0.66	1.58	0.098	-0.66	1.58	NA	-0.20	1.15	0.002
*PLEKHM3*	ENSG00000178385	2:208693026–208890284	Pleckstrin homology domain containing family M, member 3	Other	up	0.66	1.58	0.173	0.65	1.57	0.099	0.65	1.57	NA	0.25	1.19	0.073
MRP63	ENSG00000173141	13:21750783–21753223			down	-0.43	1.35	0.170	-0.45	1.36	0.099	-0.44	1.36	0.02 *	-0.15	1.11	0.083
ANAPC11	ENSG00000141552	17:79845712–79869340	Anaphase promoting complex subunit 11	Cytoplasm	down	-0.45	1.37	0.166	-0.46	1.38	0.099	-0.46	1.38	0.026 *	-0.15	1.11	0.083
ATP5J2	ENSG00000241468	7:98923520–99063954	ATP synthase, H+ transporting mitocondrial Fo complex, subunit F2	Cytoplasm	down	-0.56	1.47	0.166	-0.57	1.49	0.099	-0.57	1.48	0.041 *	-0.18	1.13	0.119
PLP2	ENSG00000102007	X:49028272–49042845	proteolipid protein 2 (colonic epithelium-enriched)	Cytoplasm	down	-0.53	1.44	0.166	-0.54	1.45	0.099	-0.53	1.44	0.041 *	-0.18	1.14	0.096
RPS29	ENSG00000213741	14:50043389–50081390	Ribosomalprotein S29	Cytoplasm	down	-0.77	1.71	0.166	-0.79	1.73	0.099	-0.78	1.72	0.047 *	-0.24	1.18	0.167

Symbols not detected by DESEq2 are denoted in Italics.

* DEGs with an adjusted p-value< 0.05.

### Biological Interpretation of Differential expression

**Fig 3:**
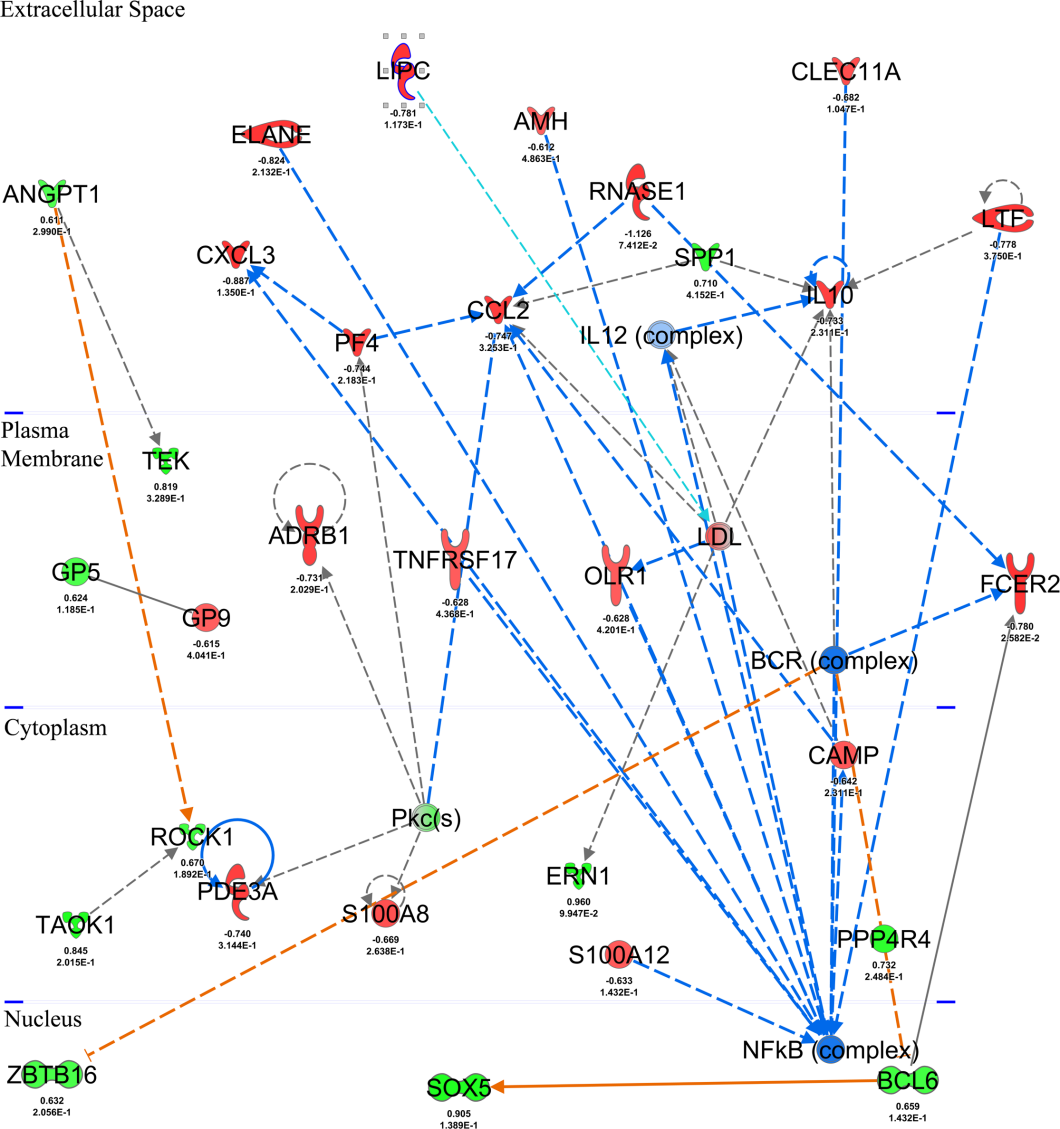
Ingenuity top gene network interaction reflecting immune response-related transcriptome changes after consumption of LGG. Nodes in the interaction network are encoded by differentially expressed genes detected by edge-R in blood from subjects consuming LGG for 28 days, up-regulated genes are depicted in shades of green and down-regulated genes are in shades of red. Transcriptional information derived from IPA knowledge database on interactions between the nodes (activation, expression, molecular cleavage or phosphorylation) was projected onto the interaction map with predicted downregulation effects represented with blue dashed lines and upregulation effects with orange lines. From this interaction map it can be seen that several downstream genes including growth factors, peptidases, G-coupled receptors and cytokines that are known to be regulated by NF-KB transcription factor are down-regulated.

The IPA pathway analysis platform was used to organize the DEG into networks of interacting genes. EdgeR, DESeq2 and TPSM-identified DEG were used for network analysis with a cutoff log_2_ ratio of ±0.6 (fold change ±1.5) to identify potentially affected biological functions and molecular networks in response to a 28-day LGG treatment. In addition, an analysis was also run using a count filter that included the maximum number of potential treatment responders (22 samples corresponding to day 28 and day 56 samples). IPA comparative analysis highlighted the similarity of overall DEGs analysis as the top molecular networks were shared among all platforms (Table 3). The highest IPA network score corresponded to edgeR results when the 22 sample filter was applied. Lower scores with less focus molecules were generated from TSPM results. Molecular networks with scores > 20 (p-value<1E -20), involving processes such as *Cellular movement*, *Immune Cell Trafficking*, *Hematological system development and function*, *Cell to Cell Signaling and Interaction*, and *Inflammatory Response*, were identified as the top common networks in response to LGG treatment (Table 3). The molecular network with the highest score (46) related to *Cell to Cell Signaling and Interaction* and *Inflammatory response* included the top down-regulated DEG identified across platforms, *FCER2 (CD23*) (FDR adjusted p<0.05) (Fig 3) that encodes the low affinity transmembrane glycoprotein receptor that modulates IgE synthesis and homeostasis in B cells [49,50]. Potential stimulatory signals for *FCER2* expression from other molecules such as *RNASE1* and human BCR complex were shown to be inhibited [51]. Similarly IL-10 expression showed predicted inhibition due to potential down-regulation of *LTF*, human IL-12 complex and *RNASE 2* [51,52,53,54]. Other genes encoding the transmembrane receptors: tumor necrosis factor receptor superfamily member 17 (*TNFRSF17*), the oxidized low density lipoprotein (lectin-like) transmembrane receptor 1 (*OLR1*); extracellular enzymes: Lactotransferrin (*LTF*) and Elastase neutrophil expressed (*ELANE*); growth factor: C-type lectin domain family 11, member A (*CLEC11A*) and the S100 calcium binding protein (*S100A12*) have been associated with induction of *NF-KappaB* [55,56,57,58,59,60,61] and were also shown to be down-regulated in our data (Fig 3) and used as supporting evidence in IPA to predict a down regulation of *NF-Kappa B* when elderly subjects are treated with LGG under our experimental conditions. Genes of the Ribonuclease RNase A Family (*RNASE1 and RNASE2*), platelet factor 4 (*PF4*) and cathelicidin antimicrobial peptide (*CAMP*), known to have a direct effect on the expression of pleiotropic monocyte chemo attractant protein chemokine C-C motif ligand2 (*CCL2*)[51,62,63,64] were also down-regulated. Thus, taken together, these IPA-based predictions support a down regulation of pro-inflammatory response linked to the inhibition of *NF-Kappa B* complex activation and inhibition of *CCL2* in response to LGG treatment.

**Table 3:** Predicted top molecular networks affected by LGG treatment after 28 day intervention.

ID	Analysis	Molecules in Network	Score	Focus Molecules	Top Diseases and Functions
1	edgeR _cpm 0.1/22	↓ADRB1,↑ANGPT1,↑BCL6,BCR (complex), ↓CAMP, ↓CCL2, ↓CLEC11A, ↓CXCL3, ↓ELANE,↑ ERN1,↓FCER2,↑GP5,↓GP9,↓IL10,IL12 (complex),LDL,↓LIPC,↓LTF, NFkB (complex),↓OLR1,↓PDE3A,↓PF4,Pkc(s),↑PPP4R4,↓RNASE1,↓RNASE2,↑ROCK1, ↓S100A8,↓S100A12,↑SOX5,↑SPP1,↑TAOK1,↑TEK,↓TNFRSF17,↑ZBTB16	46	30	Cell-To-Cell Signaling and Interaction, Inflammatory Response, Cardiovascular Disease
1	edgeR cpm 0.1/all	Akt,↑ANGPT1,↑ARR3, ↓CAMP,↓CCL2,↓CEACAM8, Cg,↓CLEC11A, ↓ELANE, ↑EP300, ERK1/2,↑ERN1,↑GP5,↓GP9,IL12 (complex),↑ITGAV,LDL, ↓LGALS1, ↓LIPC,↓LTF,NFkB (complex),↓OLR1, P38 MAPK, ↓PF4, Pkc(s), ↓PPBP, ↑PPP1R12A, ↓PRTN3, ↓RETN, ↓RNASE2, ↑ROCK1, ↓S100A8, ↓S100A12, ↑TAOK1,↑ZBTB16	42	27	Cellular Movement, Immune Cell Trafficking, Hematological System Development and Function
1	DESEq2/all	↑ADCYAP1,Akt,↑ATM,↓CAMP,↑CCR3,↓CEBPE,↓CTSC,↓CXCL3,↓ELANE,↑EP300, ERK1/2,Histone h3, ↓IFITM3,IgG,↑ITGA6,↑KMT2A, ↓LGALS1,↓LTF, ↑MDM2, ↑MICA, Mmp,↓MPO, NFkB (complex),P38 MAPK, ↑PBRM1, ↓PF4, ↓RETN, ↑ROCK1, ↑RUNX3, ↓S100A12, ↓SEMA3B, ↑SLC9A1, ↑TAOK1, ↓TRAF3IP2, ↑USP7	25	17	Cellular Movement, Immune Cell Trafficking, Hematological System Development and Function
1	TSPM/all	ADIPOQ,↓ADORA2A,Akt,↑COL3A1,↓CXCL10,↓CXCL11,↓CXCR3,↑DDX58,↓DEFB1, ↑EFEMP1,ERK1/2,↑FST,↑HMGB1,↑IFNAR1,↓IFNL1,INS,Interferon alpha, ↓LBP, ↓LILRB4, ↑MAP2K4,↑MET, P38 MAPK, PI3K (family), ↑PRL, ↓RNASE2, Rsk, ↓SCGB3A1,↑SLC30A8,↓SPSB4,↑SYK,TAC1,↑TACR1, ↑TBK1,↓TICAM1,↓VEGFA	19	10	Cellular Movement, Hematological System Development and Function, Immune Cell Trafficking
2	edgeR_cpm 0.1/22	Akt,↑ARR3,↑ATM,↑CCR3,CD3,↓CEACAM8,Cg,↑CHRNA7,↓DEFA1 (includes others),↑EP300,↑ERBB3,ERK,ERK1/2,↑ESM1,↑FGFR2,↑HAS1,Histone h3, ↑IL1RL1, ↑ITGA1,↑ITGA6,↑ITGAV,Jnk,↑KMT2A,↓LGALS1,↑MDM2,Mek,P38 MAPK, ↑PBRM1, PI3K (complex),PI3K (family),↑PRKCA,↓RETN,↓SCGB3A1,↓SFTPD,↑SMN1/SMN2	32	24	Cellular Movement, Infectious Disease, Cardiovascular System Development and Function
2	edgeR_cpm 0.1/all	↑AKT1,↑APAF1,↓AZU1,↑CD163,↓CFD,↓COMMD6,↓CXCL3,↑CXCL5,↓CXCL9, ↓E2F1,↑HIVEP2,↓HP,IGHE,Ikb,↓IL6,↑KMT2E,↓LCN2,↓LGALS3,↓LTF,↑MCM3,mir-145,↑OSM,↓PPBP,↑RELA,↓RETN,↓RNASE2,↓ROMO1,↓S100A8,↓SFN,↓TCL1A, ↓TGFB1,↓TLR7,↓TNF,↑TP53BP2,↑XYLT1	23	16	Inflammatory Response, Cell-To-Cell Signaling and Interaction, Hematological System Development and Function
2	DESEq2/all	↓AIFM3,↑APOL6,↑ASPM,↑BCL2L11,↑BRIP1,↓CAMP,↑CASP3,CASR,↑CD163, ↓DEFA4,↓DEFA1 (includes others),↓FASLG,↑FOXO1, ↑FPR2,↓HP,IL6,IL25,↓IL32, IL17F, ↑IL1B, lymphotoxin-alpha1-beta2,mir-145,↓MMP8,↑MYEF2,↓PF4, ↑PRKCB,Pro-inflammatory Cytokine, ↓PRTN3, ↑RNF19A, ↓S100A8, ↓S100A9, ↓SFTPD, ↓TGFB1, ↓TNFRSF12A, ↑XYLT1	20	16	Inflammatory Response, Cellular Movement, Hematological System Development and Function
2	TSPM/all	LMX1B↑,NRXN1↑	2	1	Cardiovascular System Development and Function, Cellular Assembly and Organization, Cellular Development

**Fig 4:**
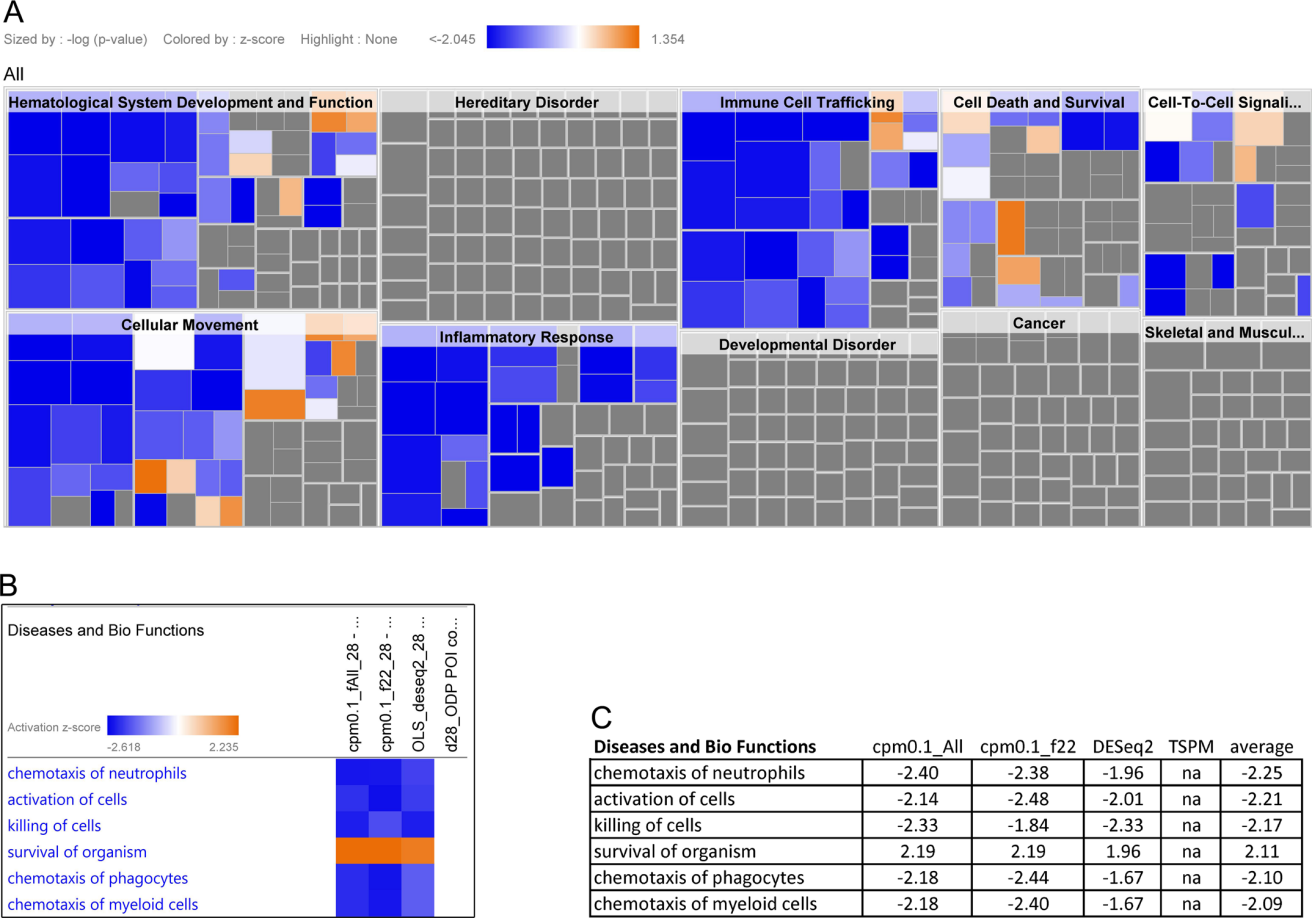
Downstream effect analysis (DEA) on whole blood cells of subjects consuming LGG for 28 days. (A).The visualization is a hierarchical heat-map generated from edgeR analysis with filtered data where the major boxes represent a family (or category) of related functions. Each individual colored rectangle is a particular biological function or disease and the color indicates its predicted state: Increasing (orange), or decreasing (blue). Darker colors indicate higher absolute Z-scores. In this view the size of the rectangle is correlated with increasing overlap significance (p-value). The image has been cropped for better readability. (B) Heat-map comparison of Diseases and Biofunctions affected across all 4 analysis (edgeR 0.1 cpm/all, edgeR 0.1cpm/ 22, DESEq2, TSPM). Similarly color represents predicted state. (C). Individual Z-scores and mean Z-scores per each Bio Function affected. The Z-score algorithm is designed to reduce the chance that random data will generate significant predictions. Negative Z-scores indicate a down-regulation of Biofunction, positives Z-scores indicate an up-regulation of function. Absolute Z-score values higher than 2.0 can be used to make biological predictions.

A heat-map generated by the Downstream Effect Analysis (DEA) tool within IPA illustrated a common set of biological processes related to cellular movement, immune cell trafficking, hematological system development and inflammatory response that were casually affected by the up- and down-regulation of genes encountered in our datasets (Fig 4). Specific functions associated with chemotaxis of neutrophils (Z-score = -2.25), activation of cells (Z-score = -2.21), killing of cells (Z-score = -2.17), chemotaxis of phagocytes (Z-score = -2.10) and chemotaxis of myeloid cells (Z-score = -2.09) were predicted to be reduced by all analysis platforms after LGG treatment, while the survival of organisms (Z-score = 2.11) was predicted to be up-regulated (Fig 4). In order to identify upstream molecules of genes in the dataset that potentially explain the observed expression changes, the IPA’s Upstream Regulator Analysis (URA) tool was utilized to examine how many known targets of each transcription regulator were present in the datasets and also compare their direction of change (expression in the day 28 relative to day 0) in order to predict likely relevant transcriptional regulators. Transmembrane receptor CD40 (Z-score -1.87, p value = 0.02), cytokine Tumor necrosis factor (TNFa)(Z-score -1.30, p-value = 0.04) and mature miRNA-146a-5P (Z-score 1.9, p value = 1.3 x 10−^5^) were identified as putative upstream regulators based on Z-scores and associated overlapping p-values.

To relate gene expression changes to previously described functional profiles, DEG were also overlapped with 50 richly annotated gene sets from the MSigDB database (http://www.broadinstitute.org/gsea/msigdb/index.jsp) which are used as hallmark gene sets that summarize and represent specific well defined biological states or processes [35]. Our dataset presented a significant overlap with 16 down-regulated genes encoding proteins involved in oxidative phosphorylation, 7 genes encoding proteins in response to IL-2 and 5 genes coding for proteins in response to IFNg stimulation (S5 Fig). In addition, genes typically up-regulated in adipogenesis and transplant rejection were also down-regulated in our dataset, indicating that dietary consumption with *Lactobacillus rhamnosus* is predicted to induce a down regulation of genes involved in response to these biological processes. To find correlations between our intervention with *L*. *rhamnosus* and its similarity at the transcriptional level to response profiles associated with pharmaceutical and other biologically active compounds, the Connectivity map (C-MAP) database was also used [36]. C-MAP results showed that the *in vivo* transcriptome obtained after a 28-day LGG intervention shared a large similarity to the transcriptome obtained after exposing human cell lines to compounds with anti-neoplastic effects (i.e.MG-132, demecolcine, decitabine, tyrphostin), anti-inflammatory action (proteasome inhibitors MG-132 and MG-262, 1-5-isoquinolinediol) for management of hypertension (sulmazole, chlortalidone),vomit inducers (i.e. emetine, cephaeline) or compounds that control apoptosis (H-7 and other topoisomerase inhibitors) (Table 4).

**Table 4:** Connectivity-map analysis results for the interventions of healthy adults with *Lactobacillus rhamnosus* GG.

Compound (medicine)	Connectivity score	Biochemical interaction	Therapeutic usage
MG-132	1	specific proteasome inhibitor reduces degradation of ubiquitin-conjugated proteins. Activates c-Jun N-terminal Kinase (JNK1) which initiates apoptosis and inhibits NF-kB activation.	Antineoplastic, inhibit or prevent tumor proliferation, inhibits IL-1B/tumor necrosis factorα induced activation of Nuclear factor-ҝi
demecolcine	0.95	Alkaloid, inhibitis mitosis at metaphase by inhibiting spindle formation	Anti-neoplastic, improve results of cancer radiotherapy
emetine	0.93	alkaloid, protein synthesis inhibitor in eukariotic cells	Used as anti-protozoal and to induce vomiting.
1,5-isoquinolinediol	0.92	Inhibitor of PARP-1 and NOS2.	Cell neuroprotective properties. PARP and NOS2 activations are implicated in deterious inflammatory responses and suppression of their activity has been correlated with to cellular protection and survival
cephaeline	0.9	alkaloid	Induces vomiting by stimulating the stomach lining, amoebicide.
MG-262	0.89	proteasome inhibitor	MG-262 Proteasome inhibition reduces proliferation, collagen expression, and inflammatory cytokine production in nasal mucosa and polyp fibroblasts.
decitabine	0.88	antimetabolite, demethylation agent	Decitabine is an anti-cancer "antineoplastic" or "cytotoxic" chemotherapy drug.
sulmazole	0.85	A1 adenosine receptor antagonist	An imidazopyridine that is 1H-imidazo[4,5-b]pyridine which is substituted at position 2 by a 2-methoxy-4-(methylsulfinyl)phenyl group. An A1 adenosine receptor antagonist, it was formerly used as a cardiotonic agent
chlortalidone	0.84	thiazide diuretic	For management of hypertension and edema.
tyrphostin AG-1478	0.84	inhibitor of EGFR tyrosine kinase activity	Commonly use as an EGF signaling blocker. Inhibits cell proliferation and arrest cell cycle in tumor cells with overexpression of EGFR.
			
H-7	-0.98	protein Kinase C inhibitor	H-7 inhibits cell invasion and metastasis in B16BL6 cancer cells through the PKC/MEK/ERK pathway. This compound is shown to inhibit Topo I and II in murine L929 cells and induce apoptosis through PKC inhibition
Irinotecan	-0.99	alkaloid, topoisomerase I inhibitor	Anti-cancer ("antineoplastic" or "cytotoxic") chemotherapy drug. This medication is classified as a "plant alkaloid" and "topoisomerase I inhibitor
Camptothecin	-1	alkaloid	Inhibits the nuclear enzyme DNA Topoisomerases, Type I. Anti-tumor activity
tyrphostin AG-825	-1		Selective ErbB2 inhibitor, Inhibit Her-2/neu signaling and promote killing of human LNCaP, C4, and C4-2 prostate cancer cells.

## Discussion

This study provides the first transcriptomic sequencing effort to determine gene expression changes in human WBC from healthy elderly individuals after daily consumption of probiotic *Lactobacillus rhamnossus* GG-ATCC53103 (LGG). Bioinformatics analysis identified a discrete set of LGG-induced DEG in WBC of elderly patients consuming LGG that returned to baseline levels after 28 additional days without LGG consumption. Monitoring the presence of LGG-derived DNA in the feces as a measure of compliance confirmed a significant increase of LGG following 28 days of consumption and a return to baseline levels after consumption was discontinued. These data suggest a LGG-dependent modulation of the WBC transcriptome in healthy elderly humans. *Lactobacillus* species have been extensively studied for their immune modulating activities [1,8]. Different studies have shown variable effects on immunity and inflammation using a variety of *Lactobacillus rhamnosus* strains which has made a generalized interpretation of results difficult [2,4,6,65]. *L*. *rhamnosus* bacterial cells and components have been shown to interact with a wide variety of host cells present in blood and intestinal tract such as epithelial and dendritic cells, macrophages and neutrophils [10,11,66,67] resulting in the secretion of pro- and anti-inflammatory cytokines. The response of explanted human peripheral blood mononuclear cells from normal or probiotic fed humans to bacterial products and immune simulators *in vitro* [68,69,70], or studies using animal models [2,71,72] has suggested some regulatory function activated by *Lactobacillus* species for modulating immunity and inflammation. However, a more robust transcriptomic evaluation of WBC from humans consuming probiotics for a prolonged time has not been previously completed. Thus, it was the aim of this study to identify DEG in human WBC from an open label Phase I study of elderly subjects participating in daily LGG consumption for a period of 28 days followed by a period equally as long without the probiotic consumption.

Increasing sequencing depth and ever-expanding coverage of next generation sequencing technology has made RNA-Seq an attractive approach for the identification of DEG in response to several different stimuli [73,74]. Molecular profiling of circulating blood cells has been associated with physiological, toxicological and pathological events originating from different tissues and organs in the body making it a rich source for potential biomarker identification [33,75,76,77,78,79] for the evaluation of treatment responses [45,80,81]. Our study consisted of whole blood RNA samples averaging 70M reads. This degree of depth is well beyond previous recommendations of 20M reads for detection of differentially expressed genes in a species with fully annotated genome [82]. The number of biological replicates used in this study per time point (n = 11) is considered to be relatively high for achieving a statistically powerful analysis when compared to the minimum of 3–6 replicates recommended for minimal statistical inference [23,83]. Overall, more power is gained by increasing the number of biological replicates relative to technical replication and sequencing depth due to the improved estimation of sample variance [23]. Appropriate handling of RNA-Seq data is essential to account for the presence of systematic variation between samples as well as differences in library composition. There is no general consensus on which method performs best when analyzing data from human WBC generated by RNA-Seq. Selecting an optimal analysis method was a challenging task as this field of research is actively growing and ongoing efforts to assess and cross-validate the different available analysis methods are being made [23,84,85,86,87,88]. We opted to use a multiple platform approach that incorporated four of the most popular statistical methods, a practice that has been recommended in several recent RNA-Seq studies to control for false discoveries [88]. A comprehensive evaluation of these packages along with a handful of other studies that have analyzed DEG in PBMC of healthy [89] or sick subjects [45], and from isolated human B-cell subsets [90], neutrophils [91] human derived cell lines [92] or human skin biopsies[33] indicate that DESeq2 and edgeR are both well equipped to account for differences in library size and composition; features that are typical of RNA-Seq data [84]. It has been suggested that high variability between biological replicates (over-dispersion) necessitates the use of a distribution model that incorporates mean and dispersion parameters to better model the mean-variance relationship such as the negative binomial model [93], that is implemented in DESeq2 and edgeR [22]. Our data is in agreement with prior observations that show edge-R performing better when analyzing data with larger fold changes. The low expressing genes (<3 counts) that were designated as differentially expressed by edge-R, but exhibited large fold changes (>1.5 in 15 genes) likely do not have a biological significance due to their very low counts. DESeq2 treated these genes as outliers and omitted them from the analysis (Table 2). Alternatively, the TSPM package, which operates on a per gene basis and the Cufflinks module “Cuffdiff” that uses RPKM (Reads per Kilobase per million base reads) [94]transformation, partially coincided with edge-R and DESeq2 but fold changes were considerably lower or no statistical inferences could be made, likely due to differences in how these methods account for biological variability [93] Thus, only DEG data produced by edgeR and DESeq2 was further used for data mining and elucidation of affected biological pathways.

RNA-seq derived expression patterns have previously shown to provide considerable high sensitivity and accuracy and to be consistent with gene detection by quantitative PCR (QPCR) as the gold standard method for validation of changes in gene expression [48,78]. In our study, QPCR of DEG identified by RNA-seq analysis was not performed as sufficient RNA from all subjects was not available after globin depletion. However, the relatively modest changes found in gene expression were provided with biological context after they were related to functional changes that reflect which cellular pathways and processes were modulated by transcriptional networks and if these changes have any clinical or pharmaceutical relevance. DEG data was used to 1) reconstruct pathways and regulatory networks using Ingenuity pathway analysis (IPA); 2) compute overlaps with hallmark gene sets that represent specific well defined biological processes in the Molecular signature database (MSigDB) and 3) find functional connections among drug, genes and diseases using the Connectivity Map (C-MAP). Comparison of gene counts revealed distinct gene expression profiles only when day 28 samples were compared against day 0. No changes were detected when day 56 was compared to day 0 or day 28. From the 25,990 genes detected by RNA-Seq, a small subset was differentially expressed in response to LGG treatment: 0.5% (DEG = 139) and 3.8% (DEG = 987) by edge-R and DESeq2 respectively ranging from log_2_ fold change of 0.5 to 1.8 (absolute fold change 1.4–3.5)(FDR<0.1) (Table 2 and S5 Table). When we compared DEG lists generated by all platforms, the top down-regulated DEG were *FCER2*, encoding the low affinity receptor for immunoglobulin E (IgE) [49] and *LY86*, that encodes a glycoprotein physically associated with RP105 (a toll like receptor family protein) to form a RP105/MD-1 complex expressed in immune cells that has most recently been involved in the patho-physiological regulation of the innate immune system and inflammation [95]. Interestingly, consumption of LGG has been associated with reduced allergic symptoms in a randomized placebo control trial of atopic eczema in neonatal and infants [15,96] possibly by the induction of regulatory cytokines [97]. LGG has also been shown to decrease synthesis of OVA specific IgE and IgG2a levels with induction of regulatory T-cells and suppression of OVA induced airway hyper responsiveness in a murine model [65,98]. Possible mechanisms of action that have been proposed include a suppression of the Th2 response in respiratory organs mediated by probiotic induced T-regulatory cells or dendritic cells [65,99]. Based on our findings, the possibility that LGG ameliorates the allergic hypersensitivity response through the down regulation of FCER2 receptor should be considered as an alternate mechanism to explore.

An additional common pool of 93 DEG (75 down- and 18 up-regulated) identified by all platforms included several transcriptional regulators, lectins, ribosomal proteins, and receptors among several molecules with similar fold changes but different statistical significance (Table 2). Data mining of DEG by prediction of functional responses based on known molecular interactions previously published was used to understand the biological impact of LGG-induced DEG [30,33,81]. Downstream transcriptomic analysis identified myeloid cell activation, and cell chemotaxis as the prominent processes predicted to be inhibited by LGG treatment. Data mining with IPA incorporated expression of downstream target genes from experimental data and compiled knowledge or reported relationships between regulators and their known targets to infer the underlying causes of their observed transcriptional changes and likely outcomes [34]. There was consensus among the different analysis platforms on the significantly activated networks that were identified (Table 3). The genetic network with the highest score (46), identified as *Cell to cell signaling Interaction and Inflammatory response* contained a series of down-regulated genes encoding transmembrane receptors-*TNFRSF17* and *OLR1*, extracellular enzymes *LTF* and *ELAINE*, lectins CLEC11A, and binding proteins: *S100A8* and *S100A12* that have been associated with induction of transcription factor NF-KappaB and additional down regulated protein coding genes *RNASE1*, *PF4* and *CAMP* known to have a direct effect on the expression of monocyte chemoattractant, CCL2 (Fig 3). Additional biological processes identified by downstream effect analysis included the decreased activation and chemotaxis of myeloid cells including phagocytes and neutrophils and a decrease in many genes coding for pro-inflammatory chemokines: CXC-motif ligand 3(*CXCL3*), pro-platelet basic protein CXC-motif ligand-7 (*PPBP*), chemokine C-C motif ligand 2 (*CCL2*), platelet factor 4 (*PF4*); antimicrobial peptides: defensin alpha 1 (*DEFA1*), azurocidin 1(*AZU1*), cathelicidin antimicrobial peptide (*CAMP*), cathepsin G (*CTSG*); S-100 calcium binding proteins: (*S1000 A12* and *S100A8)*, and lectin galactoside-binding soluble 1 (*LGALS1*) involved in chemotaxis and activation of myeloid cells (S7 Table). The most inhibited upstream regulators of inflammation (negative Z-score) were the transmembrane receptor, CD40 and pro-inflammatory cytokine TNFa, known to be associated with the initiation of inflammation. The miRNA-146a-5p microRNA, an important negative regulator of inflammation, was also predicted to be increased (positive Z-score) [100]. Further comparison of our transcriptomic data with existing annotated gene sets from the MSigDB database also supported a down regulation of genes involved in processes like oxidative phosphorylation and response to pro-inflammatory IL-2 and IFN-g cytokine stimulation, indicating that LGG is capable of affecting genes associated with the establishment of the inflammatory response albeit a low level of induction.

Our study identified a discrete set of DEG with small changes in the WBC transcriptome of elderly subjects between the ages of 65 to 80 years consuming a daily ration of LGG for 28 days. Our analysis was based on the RNA extracted from WBC. This approach included cell analysis of neutrophils that seem to be a population particularly responsive to LGG. The anti-inflammatory activity of *L*GG on myeloid cells has been shown by inhibition of both PMA and *Staphylococcus aureus* induced formation of neutrophil extracellular traps (NETs), production of reactive oxygen species and phagocytic capacity of neutrophils while protecting against cell cytotoxicity [67]. However, these results raise intriguing questions regarding the immune modulating effects of LGG in subjects facing an infection where an inflammatory response is required. Here, we predicted functional responses based on known molecular interactions previously published, in a group of healthy elderly patients with no associated clinical effects during the intervention period [18]. The relatively modest changes in gene expression and the absence of any significant changes in clinical parameters [18] indicate that LGG is a safe product when used under the conditions delivered.

To further evaluate the biological impact of host trancriptome changes induced by 28-days of LGG consumption, we compare our *in vivo* transcriptomic changes with existing data in the Connectivity map pipeline that describes cell transcriptional responses of human cell lines to bioactive molecules that play a role in disease prevention or host immune stimulation. Interestingly, our results indicated that LGG consumption induced transcriptomic changes in WBC that mimic the response induced by proteasome inhibitors [101] which anti-inflammatory effect have been attributed mainly to attenuated activation of pro-inflammatory Nuclear Factor Kappa-light-chain enhancer of activated B cells (NF-κβ), a transcription factor that positively regulate many genes that encode pro-inflammatory cytokines [102]. A high connectivity score was also found with compounds with anti-neoplasic effects and compounds that are effective against amoebal infection and control apoptosis as previously described for human intestinal mucosa responses after short term exposure to *Lactobacillus rhamnosus* [8].

## Conclusions

The analysis of WBC may provide a more robust and comprehensive approach for detecting changes in the transcriptome of circulating inflammatory and immune cells that are also representative of other tissues sites in the body. The current study indicated that whole genome expression analysis can be used to identify important pathways, functions and networks in response to probiotic consumption in humans. Although the modulation of the WBC transcriptome by LGG was modest, the data suggested than an anti-inflammatory effect of LGG could be induced by daily probiotic consumption over a period of four weeks. The changes in gene expression and subsequent analysis of functionally related pathways indicated activation of molecular circuits that could modulate host inflammation. However, such predictions will need to be validated in future studies involving placebo-fed control groups, with consumption of LGG in the presence of a provocation such as an infection, and with the inclusion of other subject populations.

## Supporting Information

S1 Fig Study protocol. (PDF)

S2 Fig Gene Clustal analysis for Lactobacillus species tuf-gene alignment. Primers and probe against tuf-gene of *Lactobacillus rhamnossus* were designed after Clustal alignment of sequences from closely related *Lactobacillus* species.
(PDF)

S3 Fig Paired comparisons among individual transcripts reads expressed as RPKM (log 10) with different RNA input levels (100, 250, 500,1000ng). T-test was used for individual comparisons.
(PDF)

S4 Fig Venn Diagram with common DEG across edgeR, DESeq2 and TSPM after consumption of *Lactobacillus rhamnosus* for 28 days. (PDF)

S5 Fig Gene computed overlap between *Lactobacillus rhamnosus*-induced gene set and hallmark gene sets in the Molecular Signature Database (MsigDB) collection. (PDF)

S1 Table Consort Checklist. (PDF)

S2 Table Globin depleted RNA yield and quality determined by gel electrophoresis. (XLSX)

S3 Table RNA sequencing data yields after sequencing and mapping. (XLSX)

S4 Table Copies per million in each library after 0.1 cpm filter. (XLSX)

S5 Table DEG generated by DESeq2 analysis. (XLSX)

S6 Table DEG generated by TSPM analysis. (XLSX)

S7 Table Downstream effect analysis. Functions and associated genes affected after consumption of *Lactobacillus rhamnosus* for 28 days.
(XLSX)
