# Supplementary material for: Transcriptomic Profile of Whole Blood Cells from Elderly Subjects Fed Probiotic Bacteria Lactobacillus rhamnosus GG ATCC 53103 (LGG) in a Phase I Open Label Study
Source: PLoS One. 2016 Feb 9;11(2):e0147426. doi: 10.1371/journal.pone.0147426 (PMC4747532; doi:10.1371/journal.pone.0147426)
Supplement: S2 Fig — Primers and probe against tuf-gene of Lactobacillus rhamnossus were designed after Clustal alignment of sequences from closely related Lactobacillus species. (PDF) [file pone.0147426.s002.pdf]

Figure S2. Lactobacillus species tuf-gene alignment used to design *Lactobacillus rhamnosus* specific tuf-gene assay

```
A. 5'  CGGTTCTGCCTTGAAAGCACTTGAAGGCGATCCTGAACAGGAAAAGGTTATCATGGAATTGATGGATACCATCGATGAATATATCCCAACCCCCAGTTCGTGAAACC 3'
B.     TGGTTCTGCCTTGAAAGCACTTGAAGGCGATCCTGAACAGGAAAAGGTTATCATGGAATTGATGGATACTATCGATGAATATATCCCAACCCCCAGTTCGTGAAACC
C.     TGGTTCTGCTTTGAAGGCCCTTGAAGGCGATCCAGAACAGGAAAAGGTTATCATGGAATTGATGGATACCATCGATGAATATATCCCAACACCTGTTCGTGAAACA
D.     TGGTTCTGCTCTGAAGGCCCTTGAAGGCGATCCAGAACAGGAAAAGGTTATCATGGAATTGATGGATACCATCGATGAATATATCCCAACACCTGTTCGTGAAACA
```

Taxonomic Key

A. Lactobacillus rhamnosus GG (ATCC 53103, NC_017482.1), L. rhamnosus LOCK908 (NC_017491.1), L. rhamnosus ATCC 8530 (NC_017491.1), L. rhamnosus LMS2-1, (NZ_GG692960.1), L. rhamnosus Lc 705 (NC_013199.1)

B. L. rhamnosus LOCK900, (NC_021723.1)

C. L. casei ATCC 334 (NC_008526.1)

D. L. paracasei subsp. paracasei 8700:2 (NC_022112.1), L. casei LOCK919, (NC_021721.1), L. casei W56, (NC_018641.1), L. casei BD-II, (NC_017474.1), Lactobacillus casei LC2W (NC_017473.1), Lactobacillus paracasei subsp. paracasei ATCC 25302 (NZ_GG670152.1), Lactobacillus casei str. Zhang (NC_014334.1), Lactobacillus casei BL23 (NC_010999.1)

Forrward and reverse primer sequences are indicated in red, the probe sequence in blue. Mismatches are indicated as N.

Forward- L. rhamnosus tuf -222F: CGGTTCTGCCTTGAAAGCA
Reverse-L.rhamnosustuf-327R: GGTTTCACGAACTGGGGTTG
Probe-L.rhamnosus tuf-261revT(MGB): CTGTTCAGGATCGCCTTC
