# Supplementary material for: Transcriptomic Profile of Whole Blood Cells from Elderly Subjects Fed Probiotic Bacteria Lactobacillus rhamnosus GG ATCC 53103 (LGG) in a Phase I Open Label Study
Source: PLoS One. 2016 Feb 9;11(2):e0147426. doi: 10.1371/journal.pone.0147426 (PMC4747532; doi:10.1371/journal.pone.0147426)
Supplement: S3 Fig — T-test was used for individual comparisons. (PDF) [file pone.0147426.s003.pdf]

250ng vs 100ng

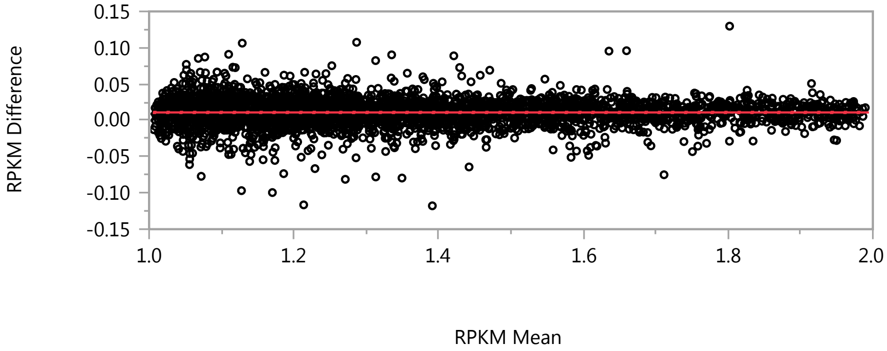

Log10(250ng-RPKM)	1.36895	t-Ratio	27.46905
Log10(100ng-RPKM)	1.35812	DF	3317
Mean Difference	0.01083	Prob > |t|	<.0001 *
Std Error	0.00039	Prob > t	<.0001 *
Upper 95%	0.01161	Prob < t	1.0000
Lower 95%	0.01006		
N	3318		
Correlation	0.99599		

500ng vs 100ng

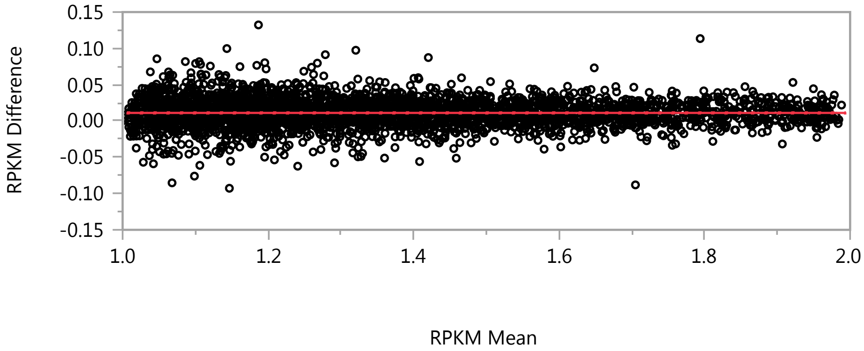

Log10(500ng-RPKM)	1.3692	t-Ratio	30.57384
Log10(100ng-RPKM)	1.35812	DF	3317
Mean Difference	0.01108	Prob > |t|	<.0001 *
Std Error	0.00036	Prob > t	<.0001 *
Upper 95%	0.01179	Prob < t	1.0000
Lower 95%	0.01037		
N	3318		
Correlation	0.99662		

1000ng vs 100ng

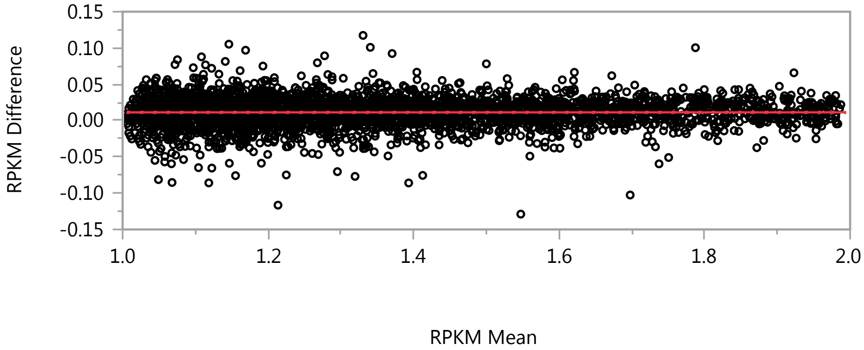

Log10(1000ng-RPKM)	1.36944	t-Ratio	29.05626
Log10(100ng-RPKM)	1.35812	DF	3317
Mean Difference	0.01133	Prob > |t|	<.0001 *
Std Error	0.00039	Prob > t	<.0001 *
Upper 95%	0.01209	Prob < t	1.0000
Lower 95%	0.01056		
N	3318		
Correlation	0.9961		

500ng vs 250ng

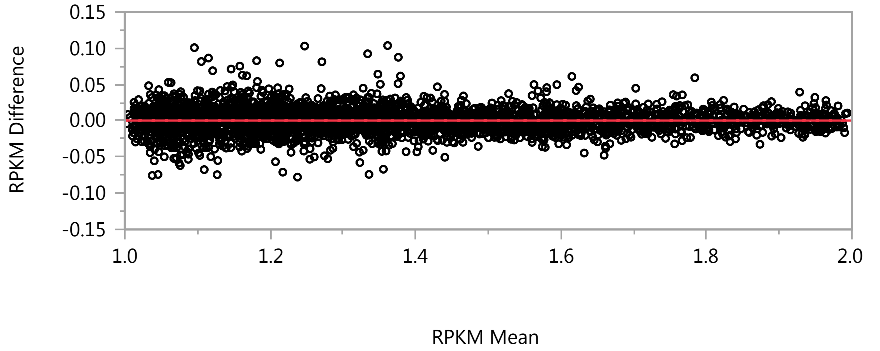

Log10(500ng-RPKM)	1.3692	t-Ratio	0.778139
Log10(250ng-RPKM)	1.36895	DF	3317
Mean Difference	0.00025	Prob > |t|	0.4365
Std Error	0.00032	Prob > t	0.2183
Upper 95%	0.00087	Prob < t	0.7817
Lower 95%	-0.0004		
N	3318		
Correlation	0.99741		

1000ng vs 250ng

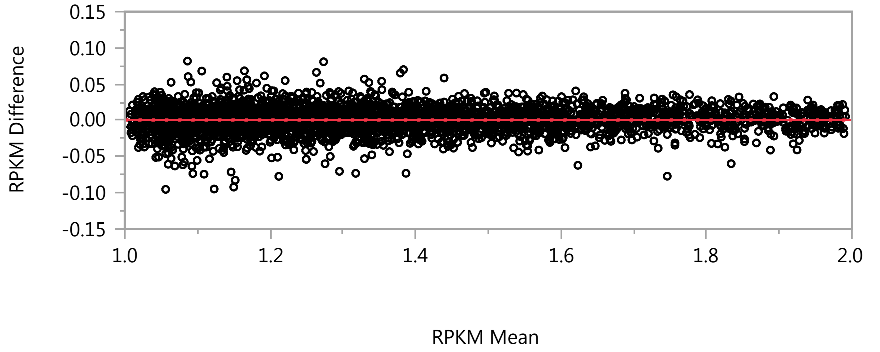

Log10(1000ng-RPKM)	1.36944	t-Ratio	1.3082
Log10(250ng-RPKM)	1.36895	DF	3317
Mean Difference	0.00049	Prob > |t|	0.1909
Std Error	0.00038	Prob > t	0.0954
Upper 95%	0.00123	Prob < t	0.9046
Lower 95%	-0.0002		
N	3318		
Correlation	0.99633		

1000ng vs 500ng

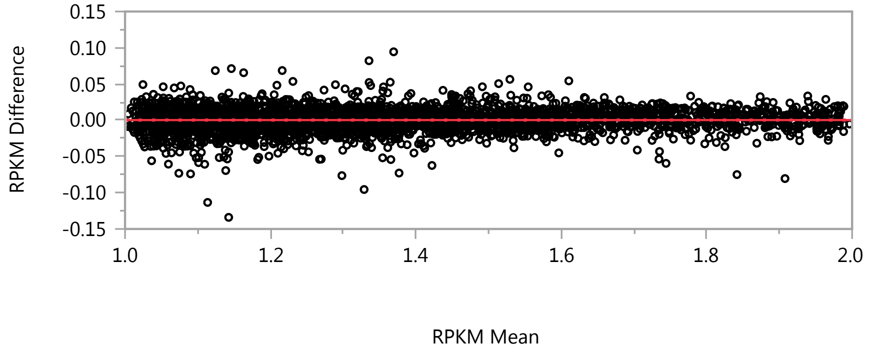

Log10(1000ng-RPKM)	1.36944	t-Ratio	0.708622
Log10(500ng-RPKM)	1.3692	DF	3317
Mean Difference	0.00025	Prob > |t|	0.4786
Std Error	0.00035	Prob > t	0.2393
Upper 95%	0.00093	Prob < t	0.7607
Lower 95%	-0.0004		
N	3318		
Correlation	0.99685		
