# Supplementary figures and images for: Transcriptomic Profile of Whole Blood Cells from Elderly Subjects Fed Probiotic Bacteria Lactobacillus rhamnosus GG ATCC 53103 (LGG) in a Phase I Open Label Study
Source: PLoS One. 2016 Feb 9;11(2):e0147426. doi: 10.1371/journal.pone.0147426 (PMC4747532; doi:10.1371/journal.pone.0147426)

Venn Diagram

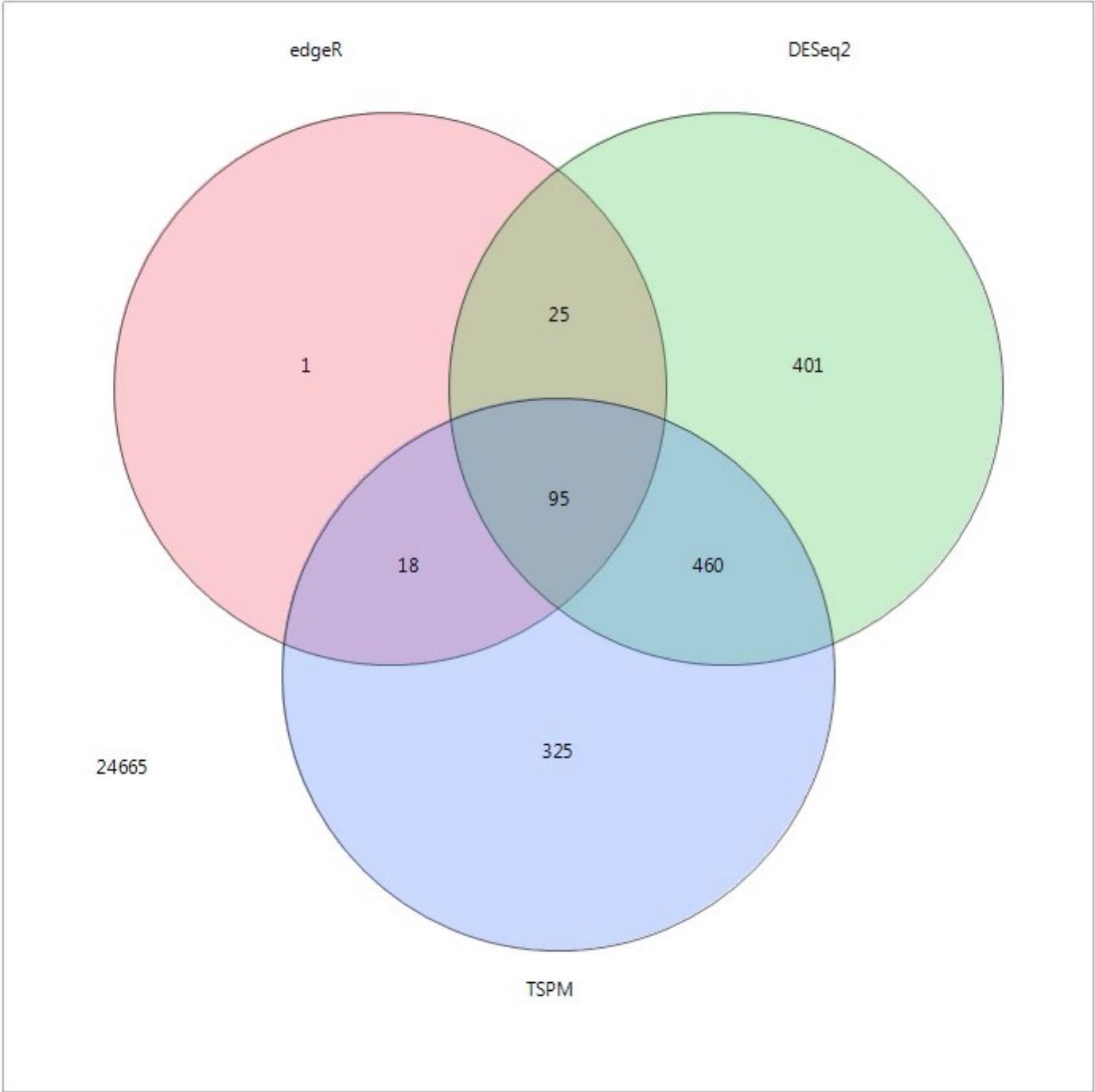

Supplement: S4 Fig — (PDF) [file pone.0147426.s004.pdf]
