# Supplementary material for: Transcriptomic Profile of Whole Blood Cells from Elderly Subjects Fed Probiotic Bacteria Lactobacillus rhamnosus GG ATCC 53103 (LGG) in a Phase I Open Label Study
Source: PLoS One. 2016 Feb 9;11(2):e0147426. doi: 10.1371/journal.pone.0147426 (PMC4747532; doi:10.1371/journal.pone.0147426)
Supplement: S5 Fig — (PDF) [file pone.0147426.s005.pdf]

Gene Set Name [# Genes (K)]	Description	# Genes in Overlap (k)	k/K	p-value	FDR q-value
HALLMARK_OXIDATIVE_PHOSPHORYLATION [200]	Genes encoding proteins involved in oxidative phosphorylation.	16		$1.49\ e^{-18}$	$7.45\ e^{-17}$
HALLMARK_IL2_STAT5_SIGNALING [200]	Genes up-regulated by STAT5 in response to IL2 stimulation.	7		$2.42\ e^{-6}$	$6.04\ e^{-5}$
HALLMARK_ALLOGRAFT_REJECTION [200]	Genes up-regulated during transplant rejection.	6		$3.1\ e^{-5}$	$5.16\ e^{-4}$
HALLMARK_ADIPOGENESIS [200]	Genes up-regulated during adipocyte differentiation (adipogenesis).	5		$3.37\ e^{-4}$	$2.81\ e^{-3}$
HALLMARK_INTERFERON_GAMMA_RESPONSE [200]	Genes up-regulated in response to IFNG [GeneID=3458].	5		$3.37\ e^{-4}$	$2.81\ e^{-3}$
HALLMARK_MYC_TARGETS_V1 [200]	A subgroup of genes regulated by MYC - version 1 (v1).	5		$3.37\ e^{-4}$	$2.81\ e^{-3}$
HALLMARK_APOPTOSIS [161]	Genes mediating programmed cell death (apoptosis) by activation of caspases.	4		$1.38\ e^{-3}$	$9.87\ e^{-3}$

Entrez Gene Id	Gene Symbol	HALLMARK_OXIDATIVE_PHOSPHORYLATION	HALLMARK_IL2_STAT5_SIGNALING	HALLMARK_ALLOGRAFT_REJECTION	HALLMARK_ADIPOGENESIS	HALLMARK_INTERFERON_GAMMA_RESPONSE	HALLMARK_MYC_TARGETS_V1	HALLMARK_APOPTOSIS	Entrez	Source	Gene Description
2879	GPX4	■	■		■			■		S	glutathione peroxidase 4 (phospholipid hydroperoxidase)
10975	UQCR11	■			■					S	ubiquinol-cytochrome c reductase, complex III subunit XI
29796	UQCR10	■			■					S	ubiquinol-cytochrome c reductase, complex III subunit X
4713	NDUFB7	■			■					S	NADH dehydrogenase (ubiquinone) 1 beta subcomplex, 7, 18kDa
9296	ATP6V1F	■								S	ATPase, H+ transporting, lysosomal 14kDa, V1 subunit F
1632	ECI1	■								S	enoyl-CoA delta isomerase 1
10476	ATP5H	■								S	ATP synthase, H+ transporting, mitochondrial Fo complex, subunit d
1327	COX4I1	■								S	cytochrome c oxidase subunit IV isoform 1
1347	COX7A2	■								S	cytochrome c oxidase subunit VIIa polypeptide 2 (liver)
1350	COX7C	■								S	cytochrome c oxidase subunit VIIc
26517	TIMM13	■								S	translocase of inner mitochondrial membrane 13 homolog (yeast)
4697	NDUFA4	■								S	NADH dehydrogenase (ubiquinone) 1 alpha subcomplex, 4, 9kDa
518	ATP5G3	■								S	ATP synthase, H+ transporting, mitochondrial Fo complex, subunit C3 (subunit 9)
65003	MRPL11	■								S	mitochondrial ribosomal protein L11
9551	ATP5J2	■								S	ATP synthase, H+ transporting, mitochondrial Fo complex, subunit F2
9927	MFN2	■								S	mitofusin 2
822	CAPG		■	■						S	capping protein (actin filament), gelsolin-like
10410	IFITM3		■			■		■		S	interferon induced transmembrane protein 3
8809	IL18R1		■							S	interleukin 18 receptor 1
9446	GSTO1		■							S	glutathione S-transferase omega 1

1690	COCH									S	coagulation factor C homolog, cochlin (Limulus polyphemus)
974	CD79B									S	CD79b molecule, immunoglobulin-associated beta
3108	HLA-DMA									S	major histocompatibility complex, class II, DM alpha
3109	HLA-DMB									S	major histocompatibility complex, class II, DM beta
3122	HLA-DRA									S	major histocompatibility complex, class II, DR alpha
6203	RPS9									S	ribosomal protein S9
9450	LY86									S	lymphocyte antigen 86
83871	RAB34									S	RAB34, member RAS oncogene family
8673	VAMP8									S	vesicle-associated membrane protein 8 (endobrevin)
10791	VAMP5									S	vesicle-associated membrane protein 5 (myobrevin)
3123	HLA-DRB1									S	major histocompatibility complex, class II, DR beta 1
10549	PRDX4									S	peroxiredoxin 4
6150	MRPL23									S	mitochondrial ribosomal protein L23
51690	LSM7									S	LSM7 homolog, U6 small nuclear RNA associated (S. cerevisiae)
6175	RPLP0									S	ribosomal protein, large, P0
6193	RPS5									S	ribosomal protein S5
10018	BCL2L11									S	BCL2-like 11 (apoptosis facilitator)
4255	MGMT									S	O-6-methylguanine-DNA methyltransferase
